# Neuronal dysfunction and gene modulation by non-coding RNA in Parkinson’s disease and synucleinopathies

**DOI:** 10.3389/fncel.2023.1328269

**Published:** 2024-01-05

**Authors:** Rosaria Meccariello, Gian Carlo Bellenchi, Salvatore Pulcrano, Sebastian Luca D’Addario, Domenico Tafuri, Nicola B. Mercuri, Ezia Guatteo

**Affiliations:** ^1^Department of Medical and Movement Sciences and Wellness, University of Naples Parthenope, Naples, Italy; ^2^Institute of Genetics and Biophysics, CNR, Naples, Italy; ^3^Experimental Neurology Laboratory, Santa Lucia Foundation IRCCS, Rome, Italy; ^4^Computational and Translational Neuroscience Laboratory, Institute of Cognitive Sciences and Technologies, CNR, Rome, Italy; ^5^Aligning Science Across Parkinson’s (ASAP) Collaborative Research Network, Chevy Chase, MD, United States; ^6^Department of Systems Medicine, University of Rome Tor Vergata, Rome, Italy

**Keywords:** ncRNA, lncRNA, miRNA, circRNA, dopamine neurons, α-synuclein, Parkinson’s disease, synucleinopathy

## Abstract

Over the last few decades, emerging evidence suggests that non-coding RNAs (ncRNAs) including long-non-coding RNA (lncRNA), microRNA (miRNA) and circular-RNA (circRNA) contribute to the molecular events underlying progressive neuronal degeneration, and a plethora of ncRNAs have been identified significantly misregulated in many neurodegenerative diseases, including Parkinson’s disease and synucleinopathy. Although a direct link between neuropathology and causative candidates has not been clearly established in many cases, the contribution of ncRNAs to the molecular processes leading to cellular dysfunction observed in neurodegenerative diseases has been addressed, suggesting that they may play a role in the pathophysiology of these diseases. Aim of the present Review is to overview and discuss recent literature focused on the role of RNA-based mechanisms involved in different aspects of neuronal pathology in Parkinson’s disease and synucleinopathy models.

## 1 Introduction

The study of how non-coding RNA (ncRNAs) contribute to neurodegenerative processes is challenging, as redundancy and limited conservation across species represent significant obstacles to this task (Hartl and Grunwald Kadow, 2013). Each subclass of ncRNAs faces different challenges when trying to establish a cause-and-effect relation in neurodegeneration. For instance, the redundancy of miRNA complicates the task of identifying specific miRNAs that contribute to the pathology. Additionally, the limited homology between species makes it difficult to model the effects of ncRNAs in simple organisms. Overall, although the role of ncRNAs in neurodegeneration is becoming increasingly recognized (Szelągowski and Kozakiewicz, 2023), their contribution to the pathophysiology of these diseases remains poorly understood. MiRNAs are short, ncRNA molecules, typically composed of 17–22 nucleotides, that play a crucial role in post-transcriptional gene regulation by either repressing translation or triggering mRNA degradation (Bartel, 2004). They have been implicated in a wide array of biological processes, including the regulation of cell proliferation, differentiation, and apoptosis (He and Hannon, 2004). Within the context of the brain, miRNAs are integral to controlling processes such as neuronal differentiation, specification, activity, and survival.

**FIGURE 1:**
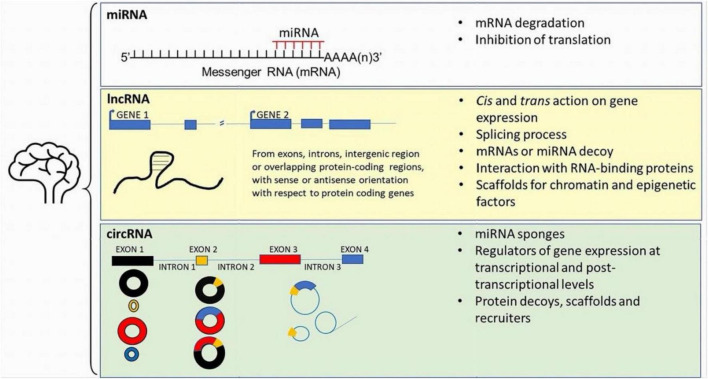
Sistematic representation of the main ncRNAs detected within the brain. See the main text for further details.

Here we will focus on few clear examples of how miRNAs and other classes of ncRNAs are linked to different aspects of neuropathology in neurodegenerative disease. Particularly, we will address the involvement of ncRNAs in Parkinson’s disease (PD) and other synucleinopathies such as dementia with Lewy bodies (DLB), multifactorial disorders characterized by accumulation of insoluble deposits which cause progressive neuronal dysfunction and ultimately neuronal loss, responsible for cognitive and motor impairments (Galvin et al., 2001). Although genetic studies have provided important information on the deregulation of gene transcripts leading to altered cellular pathways, such pathologic conditions result from complex interplay between familial predisposition, environment and aging. In this context, epigenetics appear promising to understand the complex etiology of many neurodegenerative diseases (Schaffner and Kobor, 2022). Particularly, with regard to the understanding of neuronal cell disfunction, RNA-based processes have been proposed as epigenetic mechanisms underlying local synaptic dysfunction, as RNA are transported at synapses where they regulate protein expression, synapse maturation and plastic changes. RNA-based processes include miRNA-mediated modulation of mRNA expression, as they consist of short RNA sequences that bind to 3′untranslated region (UTR) of mRNA mediating their degradation or inhibiting their transcription into proteins. Recently, studies conducted either on animal models or human brain of patients affected by neurodegenerative disease revealed significant miRNA deregulation that correlates with synaptic functions (Xylaki et al., 2023; Reviewed by Abuelezz et al., 2021). With the present manuscript we aim at providing an overview of the main classes of ncRNAs involved in PD and synucleinopathies, starting with miRNA involvement and then with lncRNA and circRNA, with the hope to shed light on their roles on neuropathology development, progression and as possible candidate biomarkers for early diagnosis and intervention in these pathologies. An overview of the ncRNAs main classes within the brain is shown in Figure 1.

## 2 Decoding the role of miRNAs in dopaminergic (DA-ergic) neurons (DAn) through knock-out (KO) models

The precise functions of individual miRNAs in brain activity remain far from being fully understood. This complexity arises from the vast number of miRNAs and the seemingly limitless combinations of miRNA-mRNA interactions. Furthermore, the strength of miRNA-mRNA binding can vary based on numerous factors, including differences in their expression levels. This variability adds an additional layer of intricacy to any attempts at functional prediction.

Compounding this challenge is the fact that a single miRNA can target multiple mRNA molecules by recognizing and binding to specific motifs known as miRNA response elements (MREs) within the mRNA’s 3′UTR. This intricate web of interactions makes it challenging to ascertain the true biological significance of a given miRNA-mRNA interaction *in vivo*. KO mouse models have been instrumental in uncovering the roles of individual miRNAs or clusters in cellular and molecular pathways. With regard to the DA-ergic system, the precise effects of miRNA deletion remain a subject of ongoing research. The influence of miRNAs on mesencephalic DAn has been extensively researched particularly in relation to their potential involvement in the development of PD. Disrupting the key miRNA biosynthetic enzyme, Dicer, leads to a distinctive morphological anomaly characterized by a failure in DAn differentiation (Huang et al., 2010). Likewise, in mature animals, a reduction in Dicer expression within the Substantia nigra (SN) is linked to deficits in motor learning and the progressive degeneration of neurons (Pang et al., 2014). This implies that miRNAs may play a role in regulating plasticity within the adult DAn system by modulating the activity of individual synapses through localized translational control (Martin and Ephrussi, 2009). Nonetheless, the specific miRNAs responsible for the physiological regulation of DAn remain uncertain.

Although KO animal models have been generated for several microRNAs only a few of them have shown a clear phenotype. An example is miR-133b a midbrain specific microRNA expressed in both mice and humans (Kim et al., 2007). miR-133b has been shown being progressively downregulated in DA-ergic deficient mouse models, leading to hypothesize for a potential role in DAn differentiation and survival through the control of the transcription factor Pitx3. However, miR-133b null mice do not show any change in mesencephalic DAn morphology and number with no differences in monoamine neurotransmission (Heyer et al., 2012). Thus, suggesting either a non-essential role of miR-133b in DA-ergic transmission or a compensatory effect exerted by a redundant miRNAs replacing miR-133b. Functional redundancy is often mediated by miRNAs having similar seed sequences thus complicating the efforts to determine the specific importance of individual miRNAs. A recent additional example of this complexity was demonstrated by the miR-34/449 family. Members of this family have been associated to the differentiation of DAn through the regulation of specific cellular pathways. An example is miR-34b/c that regulates the WNT signaling modulating DA-ergic progenitor differentiation (De Gregorio et al., 2018). However, deletion of individual family member does not show a significant phenotype suggesting that other family members might compensate for its absence. This suggests the existence of functional redundancy that makes it difficult to assign distinct roles to each individual miRNA. In these cases, only targeting the entire family unveil a clear phenotype as shown by the deletion of all the members of the miR-34/449 family (Chang et al., 2021).

Specific miRNAs, including miR-29s and miR-218s, play pivotal roles in the modulation of DA-ergic activity. While much remains to be discovered about the impact of miRNA deletion on the DA-ergic system, it is clear that microRNAs exert a profound influence on fundamental cellular processes across various types of neurons. Notably, the deletion of certain miRNAs, such as miR-29s and miR-218s, has been demonstrated to affect DA-ergic activity, leading to significant alterations in neuronal function (Pulcrano et al., 2023).

MicroRNA29 family (miR-29s) is composed of two gene clusters: miR-29a/b1 and miR-29b2/c. All the members are highly conserved and contain identical seed sequences. They are involved in multiple biological processes, including cell survival, apoptosis and inflammatory processes (Hyun et al., 2014; Adoro et al., 2015). Deletion of miR29b2/c in mice seems reducing the DA-ergic degeneration induced by MPTP injection in comparison with controls. A significative increase in the numbers of DAn, the densities of nerve terminals and the amount of dopamine (DA) were observed in miR-29b2/c KO animals exposed to MPTP in comparison with control littermates (Bai X. et al., 2021).

Similarly, miR-29a/b1 KO mice have shown a reduced vulnerability of the DA-ergic system to MPTP treatment that has been associated to a posture instability with a decreased amount of DA-ergic metabolites in the striatum (Bai et al., 2022). Both cases suggest that downregulation of miR-29s might represent a potential strategy to mitigate DA-ergic degeneration, although the exact mechanisms through which miR-29s regulate DA-ergic survival remain not completely understood. It has been hypothesized that this may happen through the release of trophic factors by both astrocytes and microglia, although the precise mechanisms have not yet been defined. Given that hundreds of genes are predicted targets of miR-29s, many of which are involved in fundamental cell survival processes it has not been possible to identify the downstream mediators of miR-29 mediated-protection (Caravia et al., 2018; Kwon et al., 2019).

More evidently, the deletion of genes coding for the two isoforms of miR-218, namely miR-218-1 and -218-2, affects DA-ergic circuitry and the release of DA, without any significant differences in the expression of DA-ergic markers (Pulcrano et al., 2023). Interestingly, miR-218 was already known as a microRNA related to motor neurons, where it controls the proper formation of the neuromuscular junction (Amin et al., 2015). Additionally, variations in miR-218 expression have been associated with amyotrophic lateral sclerosis (ALS) pathology, suggesting a potential role in the pathophysiology of the disease (Reichenstein et al., 2019). Furthermore, miR-218 has been found to promote DA-ergic differentiation (Rivetti di Val Cervo et al., 2017). KO mouse models for miR-218 have confirmed its involvement in the modulation of DAn activity (see also next section). Through the regulation of a network of synaptic-related genes, miR-218 plays a role in DA release. Its deletion leads to an upregulation of a significant number of mRNAs involved in the control of various synaptic properties, including synaptic vesicle-associated protein Sv2a and DNA-methyltransferase 3 alpha (DNMT3a), a regulator of gene expression thus leading to hypothesize a potential role of miR-218 in the epigenetic control of gene expression.

These examples highlight the challenges in pinpointing the mechanism(s) by which individual microRNAs exert their activity. While not surprising, this can be attributed to the plasticity of the nervous system, which may obscure the expected phenotype. When we consider miRNAs as an additional layer of control, complementing the translational machinery, defining their role as fine-tuners of biological processes becomes extremely challenging.

## 3 miRNA regulation of neuronal function in PD and synucleinopathy

Synucleinopathies are a group of neurodegenerative diseases characterized by accumulation of α-Synuclein (α-Syn) in the brain, which include PD, DLB and multiple system atrophy (MSA). α-Syn deposits are known as Lewy bodies (LB) and Lewy neurites (LN) in PD and DLB, and as glial/neuronal cytoplasmic inclusions in MSA (Galvin et al., 2001; Sasaki et al., 2002). In PD, the progressive degeneration of the DA-ergic nigrostriatal pathway results in brain DA depletion, an imbalance of neuronal circuits within the basal ganglia that ultimately cause typical motor and non-motor symptoms of the disease (Singh et al., 2016). In DLB, neuropathology mainly occurs in the neocortex and cognitive decline precedes motor disturbance (Dauer and Przedborski, 2003; Mayo and Bordelon, 2014). Neuropathology occurring within the SN and neocortex has been proposed to be related to the proteinaceous deposits, which affect neuron mitochondrial function (Devi et al., 2008; Nakamura et al., 2008; Shavali et al., 2008; Zhang et al., 2008; Subramaniam et al., 2014; Deas et al., 2016), somatodendritic morphology and electrical activity (Guatteo et al., 2022; Ledonne et al., 2023). However, despite intense research we still lack a complete understanding of the molecular mechanisms linking proteinaceous deposits to neuronal demise. Abnormal neuron function appears as an early sign of disease onset in synuclein PD models, occurring early and preceding motor impairments and neuronal loss typical of later stages of the disease (Tozzi et al., 2021; Ledonne et al., 2023).

Several evidence indicate the synapses located at distal sites as the neuronal compartment firstly affected by α-Syn deposits (Kramer and Schulz-Schaeffer, 2007; Schirinzi et al., 2016; Szego et al., 2019), and synaptic impairment has been related to the process of LB formation, rather than to simple fibril formation in PD model (Mahul-Mellier et al., 2020). To this regard, excitability and receptor functions at somatodendric compartment of SN DAn of a spontaneous α-Syn over-expressing rat model of PD are normal, despite the release of DA within the dorsal striatum is greatly impaired, indicating that distal synapses are the vulnerable neuronal compartment early affected by α-Syn accumulation (Schirinzi et al., 2016; Guatteo et al., 2017). Toxic forms of α-Syn propagate from distal synapses to neuron cell bodies and from affected cells to adjacent cells resulting in a cascade of LB formation, cell death and brain pathology (Braak et al., 2003; Angot and Brundin, 2009; Luk et al., 2012; Steiner et al., 2018). In this scenario, many aspects of disease progression have been intensively investigated in rodent models of PD and synucleinopathy, with the aim to unravel how LB and LN impact neuronal morphology and function, neuroinflammation and oxidative stress.

Among the molecular mechanisms causing synaptic and neuronal dysfunction in neurodegenerative diseases characterized by synucleinopathy, RNA and miRNA have been proposed, because these are small molecules, permeable to brain blood barrier (BBB), transported to synapses, where they regulate protein synthesis and participate to active synapse remodeling. Interestingly, α-Syn directly modulates miRNA levels (Xylaki et al., 2023) and miR-101a-3p is increased in the neocortex of DLB patients and correlates with synaptic plasticity impairments (Xylaki et al., 2023). Additionally, α-Syn-encoding gene *SNCA* is targeted by brain enriched miR-7 and miR-153 that bind its 3′UTR (Doxakis, 2010). It has been reported that circulating miR-153 and miR-223 are down regulated in a mouse model of PD (Cressatti et al., 2019) and this correlates with peripheral α-Syn accumulation, suggesting that miR-153 directly regulates α-Syn synthesis in mice, as previously reported in HEK293 cells (Doxakis, 2010). Reduced miR-7 levels are reported in PD patient and animal models’ brain tissue, and correlate with α-Syn accumulation (reviewed by Zhao et al., 2020). Neuropathology in PD and synucleinopathies also depends on neuron-glia interaction in which glial proinflammatory factors are released and contribute to initiate apoptotic pathways, eventually leading to DAn loss (Yang et al., 2023). Toxic metabolites as well as neurotransmitters and neurotrophic factors produced and released by activated astrocytes affect neurons at the receptor, ion channel, gene transcription levels (Bai Y. et al., 2021). In this context, many miRNAs are expressed by astrocytes, that are directly involved in development, proliferation and expression of genes regulating the inflammatory response (Neal and Richardson, 2018). Several studies have recently analyzed miRNA expression profile in cerebrospinal fluid (CSF) of early stage PD patients *vs.* healthy subjects and identified a miRNA-based biomarker panel with high predictive value (Arshad et al., 2017; Dos Santos et al., 2018). On the other hand, as PD strongly correlate with age, age-related modifications of specific miRNA may compromise the function of aged DAn, suggesting that pharmacological manipulation of miRNA levels may protect DAn in PD (Chmielarz et al., 2017).

Neuronal excitability depends on both intrinsic membrane properties (linked to ion channel/receptor function and expression levels) and strength of excitatory/inhibitory synaptic inputs they receive. Several reports suggest miRNA involvement in the regulation of ion channels expression/function in different neuronal populations. With regard to basal ganglia circuitry that is specifically involved in PD, it has been reported that in mouse striatal neurons, miR-128 suppresses the expression of several ion channels and transporters (voltage-gated sodium and calcium channels, GABA transporter and high affinity glutamate receptors) with heavy impact on animal behavior. Indeed, reduction of miR-128 causes motor hyperactivity in mice whereas its overexpression alleviate motor abnormalities in PD-like disease model (Tan et al., 2013). MiR-34b/c is an important regulator of DAn differentiation, as it increases the efficiency of fibroblast reprogramming into excitable and functionally active DA neurons, representing an important tool for regenerative medicine (De Gregorio et al., 2018). Indeed, miR-34b/c causes the appearance of voltage-gated Na^+^ and K^+^ currents and the generation of spontaneous firing in induced DAn (iDAn) (De Gregorio et al., 2018). MiR-34 also negatively controls the expression of human *ether-à-go-go* K^+^ channels in SH-SY5Y cell line (Lin et al., 2011) and inhibition of miR-34a resulted neuroprotective in rotenone-treated SH-SY5Y cell model of PD (Horst et al., 2017), suggesting the importance of this ion channel family in preserving SH-SY5Y cells when challenged with PD-inducing toxins. A direct demonstration of miRNA regulation of basal ganglia circuitry has been recently reported by Pulcrano et al. (2023), showing that miR-218 controls either intrinsic excitability of native DA neurons in adult mice, or the ability to release DA in the dorsal striatum by modulating a group of targets associated with PD and DA release at synapses (Dunn et al., 2017). Indeed, reduced expression of both miR-218 isoforms (1 and 2) causes hyperexcitability of DAn (Pulcrano et al., 2023), possibly as a consequence of Ca^2+^-activated K^+^ conductance inhibition, that limits the action potential afterhyperpolarization phase. When combined to specific transcription factors, miR-218 reprograms adult striatal astrocytes into iDAn, that are excitable and able to ameliorate motor impairments in a mouse PD model (Rivetti di Val Cervo et al., 2017). Interestingly, the expression of the voltage-dependent anion-selective channel protein (Vdac1) is increased in 6-OHDA lesioned striatum of rats trained for aerobic exercise and this was related to a decrease of miR-324 expression (Liu et al., 2019). MiRNA regulation of neuronal excitability has been also reported in SH-SY5Y neuroblastoma cells treated with 1-methyl-1,2,3,6-tetrahydropyridine/N-methyl-4-phenylpyridinium (MPTP/MPP^+^), an *in vitro* model of PD. Upon toxin exposure, TRPM2 channel expression increases in neurons both *in vitro* (Ding et al., 2019) and *in vivo* (Sun et al., 2018). TRPM2 are member of the large TRP family, a group of calcium-permeable channels activated by different stimuli such as temperature (Guatteo et al., 2005), oxidative stress and TNF-α. Interestingly, *TRPM2* gene harbors a predicted binding site for miR-625 at 3′UTR (Ding et al., 2019). Oxidative stress, induced by cell exposure to MPTP/MPP^+^, causes calcium overload through TRPM2 channel opening that contribute to neuronal demise; indeed, downregulation of TRPM2 channels prevented cell apoptosis, reactive oxygen species (ROS) generation and release of inflammatory factors. Increase of TRPM2 channel expression is mediated by lnc-p-21sponging miR-625 (Ding et al., 2019). In PC12 cells, another member of calcium-permeable TRP channels, the transient receptor potential melastatin 7 (TRPM7) is targeted by miR-22, whose over-expression significantly decreases apoptosis and ROS production following 6-OHDA exposure of PC12 cells (Yang et al., 2016). Another interesting study linked the expression of the energy sensor Kir6.2, an ATP-dependent K^+^ channel abundantly expressed in DA neurons, to elevation of miR-133b- and miR-181a-dependent inhibition of DA neuron proliferation and differentiation in a chronic MPTP/probenecid PD mouse model (Zhou et al., 2018). This report postulates that deletion of Kir6.2 in DA neurons would promote cell survival and neurodifferentiation by downregulating miR-133b and miR-181a. Also miR-96, a member of the miR-183 family that function as an oncogene, is involved in DA cell survival in PD models, by activating MAPK-signaling pathways and apoptosis. MiR-96 may directly bind to the voltage-gated calcium channel auxiliary subunit gamma 5 (CACNG5, Dong et al., 2018). In conclusion, several miRNAs have been identified as direct or indirect regulators of ion channel expression/function in native or induced DA neurons, and cell lines, and their expression levels are altered in disease models. Conversely, peculiar ion channels expressed by DA neurons regulate miRNA levels and the targeted cellular pathways. We summarized main types of ion channel expression/function regulated by miRNAs in Table 1.

**TABLE 1:** List of main miRNAs regulating ion channel expression/function in midbrain dopaminergic neurons, differentiating precursor cells and cell lines used to model PD.

Type of miRNA	Target ion channel(s)	Change of expression/function	Cell type/brain area	Main effect	References
miR-128	Na_*V*_, Ca_*V*_, GABA transporter and high affinity glutamate receptors	↓Expression	Striatal neurons	Motor behaviour (mice)	Tan et al., 2013
miR-34b/c	Na_*V*_, K_*V*_	↑Expression	Fibroblast differentiation to iDA	Increase excitability	De Gregorio et al., 2018
miR-34a	HERG K	↓Expression	SH-SY5Y cell line	Rotenone sensitivity	Lin et al., 2011
miR-218	K_*CA*_	↑Function	Native DA neurons	Excitability/DA release	Pulcrano et al., 2023
miR-218	Na_*V*_, K_*V*_, HCN	↑Expression	Astrocyte differentiation to iDA	Excitability	Rivetti di Val Cervo et al., 2017
miR-324	Vdac1	↓Expression	Striatal neurons	Ca^2+^-regulation of signaling pathways	Liu et al., 2019
miR-625	TRPM2	↑Expression	SH-SY5Y cell line	Ca^2+^ influx	Ding et al., 2019
miR-22	TRPM7	↓Expression	PC12 cell line	Ca^2+^ influx	Yang et al., 2016
miR-133b miR-181a	K_*ATP*_	↑Expression	NSC differentiation to DA neurons	Cell survival and differentiation	Zhou et al., 2018
miR-96	CACNG5	↓Expression	Native DA neurons	Cell apoptosis	Dong et al., 2018

## 4 LncRNA in α-synucleinopathies

LncRNAs are RNA transcripts longer over than 200 bp that do not encode for protein products. They are transcribed from different regions of the genome, like the intronic region of protein-coding genes, or the intergenic region; when transcribed from protein-coding genes, lncRNAs can partially overlap gene exons and have sense or antisense orientation versus the corresponding protein-coding gene (Modarresi et al., 2012; Kopp and Mendell, 2018; Bohnsack et al., 2019). LncRNAs can affect the expression of nearby genes (*cis* action) or can modulate the transcription of genes, or other cellular functions, far away a specific gene locus (*trans* action) (Kopp and Mendell, 2018). They can regulate splicing process, decoy mRNAs or miRNA, form complexes with RNA-binding proteins modulating their function, or act as scaffolds for chromatin and epigenetic factors contributing to the epigenetic modulation of gene expression (Kopp and Mendell, 2018; Kyzar et al., 2022). In recent years, lncRNAs were particularly studied for their role in the regulation of transcription and translation in physiological conditions and disease. Since lncRNA are particularly expressed and conserved in the brain (Ponjavic et al., 2009; Chodroff et al., 2010; Barry, 2014; Yang et al., 2021; Kyzar et al., 2022), their role was investigated in the epigenetic adaption of gene expression in drug addiction, aging, neuroinflammation, brain injury, neurodegenerative and neurodevelopmental diseases (Wang et al., 2018; Srivastava et al., 2021; Sivagurunathan et al., 2022; Yang K. et al., 2022; Mazzeo et al., 2023; Ruffo et al., 2023; Sherazi et al., 2023). The expression rate of lncRNAs is epigenetically modulated. In fact, changes in the main epigenetic marks (i.e., DNA methylation status at specific GpG sites) and chromatin remodeling via the modification of histone protein (H) tails at specific lysine (K) (e.g., H3K27ac, H3K4me3, and H3K36me3, H3K27me3) can exert positive or negative effects on the production of specific lncRNA (Sati et al., 2012); conversely, DNA methylation analyses revealed that DNA methylation levels of cytosine (5-mC) within the CpG islands particularly increased downstream the transcription start site of the lncRNA (Sati et al., 2012). Recently, the antisense lncRNAs, that limit the expression of the corresponding sense mRNA (Modarresi et al., 2012) or recruit transcriptional factors in brain development (Bond et al., 2009) have gathered attention in α-Syn related diseases. *SNCA*, the gene encoding for α-Syn, possesses approximately 20 CpG islands within the promoter and the first intron and is epigenetically modulated (Guhathakurta et al., 2017, 2021 and 2022). In fact, in the DA-ergic neuronal cell line, ReNcell VM, the ten-eleven translocation methylcytosine dioxygenase 1 (TET1), a CpG island binding protein capable to inhibit transcription by occupying hypomethylated CpG-rich promoters, acts as a repressor for *SNCA* by binding the intron 1 regions of the gene (Guhathakurta et al., 2022). Furthermore, in post-mortem brains of controls and PD patients, H3K4me3 resulted the only chromatin mark significantly higher at the *SNCA* promoter of the SN of PD patients both in punch biopsy and in NeuN-positive neuronal nuclei samples (Guhathakurta et al., 2021). Hence, both DNA methylation status and post-translational modulation of the histone landscape can affect the expression rate of *SNCA*.

Interestingly, an antisense gene to *SNCA* named *SNCA-AS* and producing a lncRNA has been identified in human (Fagerberg et al., 2014) and associated with hereditary neurodegenerative diseases (Zucchelli et al., 2019) and DLB (Chia et al., 2021). Recently, Rey et al. (2021) first reported that the overexpression of *SNCA-AS* in SH-SY5Y cells increased the expression of *SNCA* and the production of α-Syn; then, by RNA-sequencing analysis they investigated the molecular pathways related to both *SNCA-AS* and *SNCA* overexpression, focusing on DA-ergic and GABA-ergic synapses, for their relevance in PD and senescence. An overlapping action of *SNCA* and *SNCA-AS* on genes involved in neuronal senescence, neurite extension or synaptogenesis was reported (Rey et al., 2021) suggesting significant roles in PD and brain aging.

RNA sequencing related technologies and arrays allowed the detection of hundred deregulated lncRNAs in PD models and bioinformatics resulted useful to predict the possible target pathways (Chen N. et al., 2021; Xin and Liu, 2021; Kyzar et al., 2022; Zhang et al., 2022). We summarize in Table 2 the main validated lncRNA/miRNA pathways in *in vivo* and *in vitro* PD models.

**TABLE 2:** Validated lncRNA/miRNA pathways in *in vivo* and *in vitro* PD models.

LncRNA	Target miRNA	Pathway	PD MODEL	Effects	References
*GAS5*	miR-150	FOSL1/PTEN/AKT/mTOR	MPP^+^ treated SH-SY5Y and N2a cells	Apoptosis and neuronal activity	Ma et al., 2022
*HOTAIR*	miR-126-5p	RAB3IP	*In vivo* *In vivo*	Apoptosis and α-Syn-positive cells	Lin et al., 2019
*HOXA11-AS*	miR-124-3p	FSTL1	MPTP induced PD mice MPTP treated SH-SY5Y cells LPS induced BV-2	Neuroinflammation and neuronal apoptosis	Cao et al., 2021
*MALAT1*	miR-135b-5p	GPNMB	MPP^+^ SK-N-SH and SK-N-BE cells	Proliferation and apoptosis	Lv et al., 2021
	mir-124	??	MPTP induced PD mice MPP^+^ treated SH-SY5Y cells	Apoptosis	Liu et al., 2017
*MIAT*	miR-221-3p	TGFBR1/TGF-β1/Nrf2	MPTP-induced PD mice MPP^+^ treated MN9D cells	neuronal injury	Lang et al., 2022
*NEAT1*	miR-124-3p	PDE4B	MPP^+^ treated SH-SY5Y cells	Cell viability, cytotoxicity and inflammation	Chen M. Y. et al., 2021
	miR-124	KLF4	MPP^+^ treated SH-SY5Y cells	Apoptosis	Liu et al., 2020
*NORAD*	miR-204-5p	SLC5A3	MPP^+^ treated SK-N-SH and SK-N-AS cells	Neuroprotection	Zhou S. et al., 2020
*OIP5-AS1*	miR-137	NIX	MPP^+^ treated SH-SY5Y cells	Mitochondrial autophagy	Zhao et al., 2022
*SNHG1*	miR-216a-3p	BAX	MPP^+^ treated SK-N-SH cells	Apoptosis	Wang H. et al., 2021
*SNHG7*	miR-425-5p	TRAF5/NF-κB	Rot-mediated PD rat SH-SY5Y cells	Inflammation and oxidative stress	Zhang et al., 2021
*SNHG14*	miR-135b-5p	KPNA4	MPP^+^ treated SK-N-SH cells	Neuronal injury	Yuan et al., 2022
*TUG1*	miR-152-3p	*PTEN*	MPTP-induced PD mice MPP^+^ treated SH-SY5Y cells	Apoptosis, oxidative stress, and neuroinflammation	Zhai et al., 2020
*UCA1*	miR-651-5p	KPNA4	MPP^+^ treated SH-SY5Y cells	Apoptosis, inflammation, oxidative stress and neuronal damage	Hao et al., 2023

AKT, protein kinase B; BAX, Bcl2 associated X trotein; FOSL1, Fos ligand1; FSTL1, Follistatin-like 1; GAS5, growth arrest-specific 5; GPNMB, glycoprotein non-metastatic melanoma protein B; HOTAIR, HOX Transcript Antisense Intergenic RNA; HOXA11-AS, Homeobox A11 antisense RNA; KLF4, Kruppel-like factor 4; KPNA4, karyopherin subunit alpha 4; NF-K b, nuclear factor kappa-light-chain-enhancer of activated B cells; MALAT, metastasis-associated lung adenocarcinoma transcript 1; MIAT, myocardial infarction-associated transcript; MPP^+^, N-methyl-4-phenylpyridinium; MPTP, 1-methyl-4-phenyl-1,2,3,6-tetrahydropyridine; m-TOR, mammalian target of rapamycin; *NEAT1*, nuclear enriched abundant transcript 1; Nrf2, nuclear factor E2-related factor 2; NORAD, RNA Activated by DNA Damage; OIP5-AS1, Opa Interacting Protein 5antisense RNA; PDE4B, phosphodiesterase 4B; PTEN, Phosphatase and tensin homolog; RAB3IP, RAB3A Interacting Protein; SLC5A3, solute carrier family 5 member 3; SNHG, small nucleolar RNA host gene; TGF-β1, transforming growth factor-β1; TGFBR1, TGFB receptor 1; TRAF5, tumor necrosis factor receptor-associated factor; TUG1, taurine upregulated gene 1; UCA1, urothelial carcinoma-associated 1.

## 5 CircRNAs in α-synucleinopathies

CircRNAs are a large class of ncRNA generally rising from back splicing of linear pre-RNA of protein coding genes. As a consequence, they result from the fusion of either exons, introns, UTR, or both exon-intron into covalently closed loops (Lu et al., 2019; Lu, 2020; Dorostgou et al., 2022). Due to their closed-loop structure that lacks free 5′ and 3′ ends, these molecules are highly stable and preserved from RNAse degradation. Over 20000 circRNA have been detected in eukaryotic cells, mainly within the cytoplasm, but also in nuclear compartment. Their expression is tissue-specific, age-related, developmental-stage related and epigenetically modulated (Rybak-Wolf et al., 2015; Mahmoudi and Cairns, 2019; Abbaszadeh-Goudarzi et al., 2020; Han et al., 2020; Hanan et al., 2020; Doxakis, 2022). Usually, cirRNAs do not code for proteins; conversely some of them contain internal ribosome entry site (IRES), hence potentially maintain the ability to be translated into protein independently of the 5′cap structure critical for the initiation of translation (Li J. et al., 2020). Alternatively, adenosine methylation (m^6^A) is widespread on exon-derived circRNAs (Zhou et al., 2017) and also promotes protein translation in a cap-independent manner (Wang et al., 2015), particularly under environmental stress (Yang et al., 2017; Chen N. et al., 2021). In general, circRNAs act as miRNA sponges, thus sequester target miRNAs through a specific miRNA response element (MRSe), and inhibit the miRNA suppressive effect on translation or the miRNA dependent degradation of mRNAs (Lu et al., 2019; Lu, 2020; Dorostgou et al., 2022). In the nucleus they regulate gene expression at transcriptional and post-transcriptional levels by binding RNA binding proteins and modulating selective splicing; emerging evidence has revealed that a group of circRNAs can also serve as protein decoys, scaffolds and recruiters (Zhou W. Y. et al., 2020; Yang L. et al., 2022).

Atlas of CircRNA expression in the brain revealed that in human and mouse these ncRNAs are highly abundant, conserved, dynamically expressed and often differentially expressed compared to their linear mRNA isoforms; since their stability and the low division rate of neurons, they also tend to accumulate in the brain (Rybak-Wolf et al., 2015). However, transcriptome studies and RNA sequencing followed by bioinformatic approach provided insights on the activity and the functional roles of circRNAs in the brain revealing involvement in aging, neurodevelopment, neurogenesis, angiogenesis, neuronal plasticity, autophagy, apoptosis, and inflammation; circRNAs altered expression in disease like brain-tumor growth, traumatic brain injury or acute and chronic neurodegenerative disorders has been reported (Lukiw, 2013; Chen et al., 2016; Errichelli et al., 2017; Huang et al., 2018; Xu et al., 2018; Dube et al., 2019; Mehta et al., 2020; Li et al., 2023). In this respect, studies on the involvement of circRNA in PD are promising for the pathogenesis, diagnosis, and treatment of the disease (Dorostgou et al., 2022; Doxakis, 2022).

Since the main hallmark in PD is the aggregation of α-Syn in LB, the modulation of *SNCA* gene by circRNAs is particularly intriguing. The accumulation of α-Syn in the SN of PD patients parallels the decreased expression of miR*-7* (McMillan et al., 2017) and the Cerebellar degeneration-related protein 1 antisense RNA (CDR1as), also known as the circular RNA sponge for miR-7 (ciRS-7), is the “super sponge” of miR*-7*, due to more than seventy binding sites for miR7 in its approximately 1500 nt long sequence (Zhang and Xin, 2018). Hence, ciRS-7 is involved in the upstream regulation of α-Syn sponging miR*-7* that by itself can directly block the expression of *SNCA* in both mouse model of PD and SH-SY5Y cells (Junn et al., 2009). Similarly, in zebrafish model, miR*7* silencing share the same phenotype of ciRS-7 injection (Peng et al., 2015; Piwecka et al., 2017). Interestingly, the silencing of ciRS-7 can occur through the recruitment of Argonaute 2 (Ago2) protein and the interaction between ciRS-7 and miR-651-5p (Hansen et al., 2011), a ncRNA downregulated in PD patients with several targets in the brain related to neurological disease, included *SNCA* (Uwatoko et al., 2019). Hence, a circuitry involving the interaction of ciRS-7 with miR-7 or miR-651-5P may result critical for PD induction or suppression.

Interestingly, the *SNCA* mRNA itself has two circRNA forms originating from the coding region (i.e., Hsa_circ_0070441) and the 3′UTR (i.e., hsa_circ_0127305, circSNCA). In SH-SY5Y cells *SNCA* mRNA and circSNCA compete for the binding of miR-7 and circSNCA increased the expression rate of *SNCA* just sponging *miR-7* as a competitive endogenous RNA (ceRNA). In parallel the expression rate of apoptosis and autophagy markers also correlates to circSNCA expression in PD (Sang et al., 2018), providing evidence that circSNCA may represent a potential target in PD treatment.

The circular transcript of the pantothenate kinase 1 (*Pank1*) gene (i.e., circ-Pank1) is highly expressed in the SN of PD model mice treated with rotenone and in cell model of DA-ergic neurons like the MN9D (Liu et al., 2022). The molecular target of circ-Pank1 is miR-7a-5p, a negative modulator of *SNCA* expression. Hence, by sponging miR-7a-5p, the circ-Pank1 contributes to the increased expression and the accumulation of α-Syn in the pathogenesis of PD. Accordingly, circ-Pank1 KO ameliorates both locomotor dysfunction and damage in DA-ergic neurons (Liu et al., 2022).

CircZip-2 is the circular form of the *Zip-2* gene (Kumar et al., 2018) encoding for ZRT/IRT-like protein 2, a transcription factor in immune response (Pukkila-Worley et al., 2012). RNAi-based silencing of *Zip-2* in PD model of *Caenorhabditis elegans* expressing the human α-SYN, reduced the aggregation of α-Syn thus leading to prolonged lifespan of the nematode. Downstream pathways involving insulin signaling pathways and Daf-16 resulted responsible for the modulation of α-Syn, the production of ROS, acethylcoline and acethylcholinesterases levels, and longevity (Kumar et al., 2018). Consistently the expression rate of circZip-2 was significantly reduced in PD model of *C. elegans*, as compared to wild-type strain and miR-60 resulted the prime target of circZip-2. Hence, Kumar and co-workers suggested that circZip-2 may be protective against PD sponging the miR-60 and that the loss of circZip-2 enhanced the miR-60 activity causing the downregulation of PD protective genes in turn; conversely, in case of *Zip-2* silencing, insulin signaling via daf-16 pathway restored PD protective activity (Kumar et al., 2018).

An additional circRNA with potential neuroprotective effects in PD is circDLGAP4. Originally discovered as RNA sponge for miR-143 in ischemic stroke outcomes (Bai et al., 2018), this ncRNA resulted down regulated in several cell models of PD, affecting mitochondrion integrity, apoptosis rate, cell viability, and autophagic flux (Feng et al., 2020). In mouse, human and MN9D and SH-SY5Y cell lines the circDLGAP4 targets miR-134-5p, a ncRNA overexpressed in PD *in vivo* and *in vitro* models. In this respect, Feng et al. (2020) revealed that the circDLGAP4/miR-134-5p pathway affects both the cAMP response element binding protein (CREB) and its downstream targets like BDNF, PCG-α (Feng et al., 2020), suggesting a neuroprotective circDLGAP4/miR-134-5p/CREB pathway in PD pathogenesis.

Also, circular RNA sterile α motif domain containing 4A (circSAMD4A) is over expressed in MPP^+^ treated SH-SY5Y cells and participates in the apoptosis and autophagy of DAn via the miR-29c-3p-mediated 5′AMP-activated protein kinase (AMPK)/mTOR pathway (Wang W. et al., 2021).

The screening of RNA-seq libraries from the amygdala, SN, and medial temporalis gyrus collected post-mortem from 42 PD patients and 27 healthy controls identified a large set of cirRNAs showing a specific expression in the different brain areas and differences in the expression levels in health and disease conditions (Hanan et al., 2020). Attention was focused on circSLC8A1, the circular form of the mRNA for the sodium/calcium exchanger solute carrier family 8 member A1 (*SLC8A1*). This circRNA has binding sites for miR-128 and is capable to bind Ago2 protein. Since the targets of miR-128 are increased in PD patients, Hanan et al. suggested that circSLC8A1 could regulate miR-128 in PD (Hanan et al., 2020). The author also demonstrated a correlation between circSLC8A1 and oxidative stress-related Parkinsonism and suggested a role for circSLC8A1 in the modulation of neuronal survival and aging (Hanan et al., 2020). However, the same study identified 3407 cirRNAs expressed in PD only and 1028 circRNA expressed in controls only, suggesting that the biogenesis of circRNAs by backsplicing could be disease-related. A similar approach was used to identify the expressed cirRNAs in in the hippocampus (HP), cerebral cortex (CC), cerebellum (CB) and striatum (ST) of MPTP-induced PD mouse model (Jia et al., 2020). This study provided a map of differently expressed circRNA in specific brain area, and resulted useful to predict PD-related signaling pathways (e.g., Mmu_circRNA_0003292/miR-132, mmu_circRNA_0001320/miR-124, and the mmu-circRNA-0003292/miRNA-132*/*NR4A2 axis) and to construct a ceRNA network, which included six circRNAs, 13 miRNAs, and 112 mRNAs (Jia et al., 2020). Hence, bioinformatics approach provided important information for further study in PD.

Lastly, the circular RNA form of IQCK, MAP4K3, EFCAB11, DTNA, and MCTP1 were identified by RNA sequencing and further validated resulting overexpressed in the white matter of the multiple system atrophy cortical tissue (Chen et al., 2016), revealing perturbation of circular transcriptome in α-synucleinopathies.

Hence, ciRS-7, circSNCA, circDLPAP4, circZip-2, circSAMD4A, cicSLC8A1 and circ-Pank1 are key circRNAs in PD (Dorostgou et al., 2022; Liu et al., 2022) with mechanisms related to: (i) *SCNA* mRNA overexpression (i.e., ciRS-7/miR-7 pathway and circ-Pank1/miR-7a-5p pathway in PD progression) (Rybak-Wolf et al., 2015; Chen and Schuman, 2016; Piwecka et al., 2017; Liu et al., 2022); (ii) autophagy suppression and apoptosis (i.e., circSNCA*/*miR-7 pathway and circSAMD4A/miR-29C-3p/AMPK/mTOR pathway in PD progression (Kumar et al., 2018; D’Ambra et al., 2019); (iii) inhibition of protective genes in PD (i.e., circZip-2/miR-60-3P pathway in PD suppression) (Kountouras et al., 2012; Gokul and Rajanikant, 2018; Vogel et al., 2018): (iv) the modulation of the RNA binding protein Ago2, that is notably involved in the degradation of mRNA target by miRNAs (i.e., ciRS-7/miR-671-5p pathway in PD suppression and circSLC8A1/miR-128 pathway effects under investigation in PD) (Hansen et al., 2011; Xu et al., 2015; Hanan et al., 2020).

## 6 ncRNA as biomarkers and therapeutic target in PD

As suggested above, also miRNA could be a suitable biomarker in the diagnosis of several diseases (Wang et al., 2016; Pogribny, 2018; Fazeli et al., 2020) because of their tissue specific expression, easy detection and stability in body fluids (Chevillet et al., 2014). In the last years many researchers supported the idea that miRNA could be useful in the diagnostic path of neurological disease. For instance, miR-218 and miR-320 were found highly expressed in depressed PD patients, providing a useful biomarker for early diagnosis of PD (Wan et al., 2023), especially because the psychiatric symptoms emerge several years before motor symptoms in patients. In this context, it would be crucial to find the same biomarker for depression and PD to develop a prevention program and an early treatment. Further to this point, Xing et al. (2020) observed that miR-218 is downregulated in prefrontal cortex of PD patients, supporting the idea that miR-218 could play an important role in aetiopathogenesis of PD. The authors further suggest that the downregulation of miR-218 together with the downregulation of miR-124 and miR-144 active nuclear factor κ light chain enhancer of activated B cells (NF-κB) have a crucial role in the pathogenesis of PD (Xing et al., 2020). Moreover, miR-144 (together with miR-199b, miR-221, miR-488, miR-544) increases in gyri cinguli of PD patients’ brains, the authors suggest that they could modulate gene expression implied in PD: *SNCA*, *PARK2* and *LRRK2* (Tatura et al., 2016).

However, the alteration in miRNAs expression is not restricted to PD but it was amply described in other neurological disease. Patients with frontotemporal dementia (FTD) show a significant downregulation of miR-663a, miR-502-3p and miR-206 in the plasma if compared to healthy people (Grasso et al., 2019). Moreover, elevated concentration of miR-520f-3p, miR-135b-3p, miR-4317, miR-3928-5p, miR-8082 and miR-140-5p were detected in CSF of prodromal Huntington’s Disease patients (HD) (Reed et al., 2018).

Overall, these results highlight the importance of miRNAs in neurological disease, not only as biomarker of pathologies but also as therapeutic target. In this regard, a piece of evidence reported that miRNAs are involved in the regulation of DA-ergic circuits, α-Syn production and animal behavior. For instance, in the PD animal model 6-OHDA, the overexpression of miR-221 -usually downregulated in patients, improved motor behavior targeting the Bim/Bax/caspase-3 signaling axis, known to be involved in the apoptosis of DA-ergic cells in SN (Yao et al., 2023). In the same PD model, the overexpression of miR-375 reduces DA-ergic damage, oxidative stress and inflammation, by the inhibition of transcription factor specificity protein 1 (Cai et al., 2020). Moreover, the up-regulation of miR-218 could reduce DA-ergic damage in SN diminishing the expression of LASP1, a component of dendritic spines and synapses (Ma et al., 2021).

Concerning α-Syn (see also sections above), miR-7 was reported to downregulate this protein in MPTP-induced PD models and prevent the accumulation (Junn et al., 2009). Further to this point, miR-181 overexpression increases the α-Syn-induced DA neuronal loss and correlates with neurotoxicity, while its inhibition exerts a neuroprotective effect (Stein et al., 2022).

As mentioned before, besides PD, miRNAs could be a suitable therapeutic target also in other neuronal diseases. To this regard, HD mice models that overexpress miR-196a in the brain showed improvements in neuropathological progression, at both cellular and behavioral levels (Cheng et al., 2013). miR-124 slows down the progression of HD possibly by increasing neurogenesis (Liu et al., 2015). Gascon et al. have demonstrated that miR-124 is involved in FTD as well, in fact the ectopic expression in the medial prefrontal cortex is able to rescue behavior in FTD mice model through the action on the AMPA receptor (Gascon et al., 2014).

It’s important to note that the three neurological diseases mentioned above share in common a dysregulation of DA circuits, regard to this for example: it is reported that miR-133b is selectively expressed in midbrain DA neurons and midbrain tissue from PD patients are deficient of this miRNA, authors suggest that it is able to modulate maturation and function of these neurons (Kim et al., 2007).

The search of ncRNAs in biological fluids, particularly miRNA, as potential prognostic and/or diagnostic biomarkers in brain diseases, recently extended to the upcoming ncRNA (Bahn et al., 2015; Li Y. et al., 2020; ; Chen Y. et al., 2021; Mahmoudi et al., 2021; Ravanidis et al., 2021; Tan et al., 2021). In this respect, circulating lncRNAs were detected in the in peripheral blood mononuclear cells (PBMCs), plasma and exosome of PD patients providing a molecular signature useful for prediction, diagnosis, prognosis and therapy in PD pathogenesis (Quan et al., 2020; Zou et al., 2020; Honarmand Tamizkar et al., 2021; Akbari et al., 2022; Huang et al., 2023; Sarıekiz et al., 2023). In addition, over a panel of eighty-seven circRNAs highly expressed in the brain, six only (i.e., circMAPK9, circHOMER1, circSLAIN1, circDOP1B, circRESP1, circHOMER1, circSLAIN1, and circPSEN1) targeting miR-516b-5p, miR-526b-5p, miR-578, miR-659-3p, and miR-1197, respectively, resulted deregulated in PBMCs collected from 60 idiopathic subjects with PD compared to 60 healthy subjects (*n* = 60) (Ravanidis et al., 2021). Interestingly, the network of the six deregulated circRNAs in PD patients includes RNA-binding proteins involved in the neurodegeneration-associated diseases like Fused in Sarcoma (FUS), TAR DNA binding protein (TDP43), FMR1, and Ataxin 2 (ATXN2) (Ravanidis et al., 2021).

A similar study was carried out by Kong et al. in 2021 (Kong et al., 2021) in 4 PD patients and 4 healthy controls demonstrating 129 circRNAs up-regulated and 282 circRNAs down-regulated in the PBMCs of PD patients. The linear form of the top 10 deregulated circRNAs included genes involved in oxidative stress response and hemostasis pathways. The ceRNA interaction network of circRNA-miR-mRNA in PD patients was also provided revealing 13 miRNAs, 10 differently expressed mRNA and 10 circRNA (Kong et al., 2021).

Lastly, Zhong et al., provided evidence that circulating circRNAs panel acts as a biomarker for the early diagnosis and severity of PD (Zhong et al., 2021) identifying and validating circFAM83H, circARID1B, circHUWE1 and circTCONS-I2-0002816 as markers of PD progression. In this respect, circARID1B and circTCONS-I2-0002816 were further validated as markers to predict PD at early stage, whereas circFAM83H, circARID1B, circTCONS-I2-0002816 and circHUWE1 could be used to discriminate between PD at early or late stages.

Recently, the screening of circRNA was carried out in the blood exosome of n.80 PD patients at pre- and after 2 weeks long rehabilitation (Duan et al., 2023). This interesting study revealed deregulated circRNA in PD patients and that the expression profiles of cirRNA further remodels following rehabilitation as an epigenetic adaptive response. Interestingly, the expression profile of hsa_circ_0001535, and hsa_circ_0000437, related to the aggregation of a-syn and neuro-inflammation via the sponging of hsa-let-7b-5p and hsa-let-7c-5p, respectively, was validated pre and after rehabilitation revealing higher expression levels in pre rehabilitation and decreased expression at post-rehabilitation, but at different degree.

Taken together, upcoming ncRNA in the blood may serve as diagnostic and prognostic biomarkers for PD.

Next step, research should focus on how miRNAs and other ncRNAs influence DA and DAn in healthy conditions and in neurological disorders, and if there are other dysregulated miRNAs specifically expressed in DAn, this demonstration would allow to develop a more precise treatment with a specific catecholaminergic target.

In conclusion each of these encouraging result support the idea that in the future we could use ncRNA drugs in clinical practice, to date we have few drugs based on RNA interference technology approved by FDA with other drugs in advanced clinical trials (for a rev on RNAi-Based Therapeutics see Traber and Yu, 2023). Our hope is that in the near future we will increase the availability of these drugs, in order to develop not only more precise therapy, but early biomarker as well in order to treat patients in early stage of the disease when we are able to preserve as many cognitive functions as possible.

## 7 Discussion and conclusion

In the present manuscript we have overviewed recent literature reporting the involvement of ncRNAs (miRNAs, lncRNAs and circRNAs) in the development and function of the midbrain DA-ergic system, relevant to healthy conditions or to PD and synucleinopathies. Despite intense research efforts, their precise role in controlling physiological functions in the brain has yet to be elucidated. ncRNAs represent a hot research topic worldwide as they may be useful tools for diagnosis, prognosis and therapeutic biomarkers for brain diseases. With regard to PD and synucleinopathy sharing the abnormal accumulation of the toxic fibrillar α-Syn in intraneuronal inclusions, although the complexity of human brain and its accessibility pose challenges for therapeutic interventions, insights gathered from studying ncRNAs could catalyze the implementation of current pharmacological strategies. Recently, there has been a proposal to downregulate α-Syn expression using miRNA-based techniques (such as miRNA-mimics or anti-miRNAs) as a promising approach to slow down the progression of Parkinson’s disease (PD) and synucleinopathies. Therefore, identifying a group of miRNAs capable of modulating the expression of genes directly involved in the etiopathology, interacting with α-Syn gene or mRNA, and inhibiting its expression, could hold relevance for potential pharmacological interventions. This has been the case for, miRNA-7 and miRNA34b/c that have been described both decreased in PD patients’ brain (reviewed by Nakamori et al., 2019; Vilaça-Faria et al., 2019) possibly leading to α-Syn overexpression. However, a higher degree of success has been achieved using RNA mimicking sequences referred to as ASOs (Antisense Oligonucleotide Sequences) to enhance neuronal regeneration *in vivo*, promoting astrocyte-to-neuron conversion in a chemical model of the disease (Qian et al., 2020). This suggests that an alternative pharmacological approach targeting the endogenous regeneration of dopaminergic innervation, through small RNA sequence, may have a considerable chance of success.

The transcriptional modulation of genes encoding for proteins involved in a common signaling pathway links the genome to specific neuron functions, either in physiological or in pathological states. In this context, the regulation of ion channels’ gene expression is a key element to understand the relationship between neuronal excitability profile and the output response produced by a specific neuron type, as this feature defines its functional identity (Schulz et al., 2006). With regard to the DA-ergic system and to DAn, we and others have reported evidence showing how their functional fingerprint is finely tuned by different miRNAs, and alteration of specific miRNA expression levels heavily impacts DAn intrinsic excitability and synaptic output (De Gregorio et al., 2018; Pulcrano et al., 2023). MiRNA dysregulation at the synaptic level has been reported in many neurodegenerative diseases characterized by cognitive impairment (Abuelezz et al., 2021) drastically affecting local protein synthesis and target gene expression.

The intricate interplay between miRNAs and ncRNA in controlling synaptic activity, neuronal excitability, and their role in the progressive degeneration of DAn remains a complex and partially answered question. While there is mounting evidence of their involvement in human brain-related diseases, specific downstream pathways still lack comprehensive understanding. This complexity arises from various cellular factors, including the number of miRNAs capable of binding identical or adjacent sequences at the 3′UTR untranslated region of a given target, their concentration, and accessibility to the binding region. Additionally, miRNAs have been reported to bind to lncRNAs, acting as sponges for specific miRNAs and effectively sequestering them, thus preventing their binding to target mRNAs. This creates a competitive scenario where miRNAs may target both mRNAs and lncRNAs with similar miRNA response elements. We have provided insight on lnc RNA, circRNA and miRNA pathways that have been predicted by bioinformatic approach and further validated in PD models to regulate the pathogenesis of PD In addition, the ability of ncRNAs to play a role in the epigenetic regulation, by guiding chromatin-modifying complexes, influencing DNA methylation, and participating in RNA interference pathways in development and various diseases, has provided insights into their potential use in therapeutic approaches and paved the way for further translational approaches.

The regulatory circuitry formed by miRNAs, lncRNAs, and mRNAs might serve as a fundamental mechanism for refining gene expression. It could potentially allow cells to adapt swiftly to changing environmental cues, ensuring that protein levels remain finely tuned to meet the demands of the moment. This delicate balance is vital for cellular functions ranging from differentiation and development to response to stress and disease. Therefore, changes in any of these influencing factors may affect a miRNA’s capacity to regulate its target(s), leading to phenotypic individual differences that might vary depending of the genetic background or the environmental condition.

Handling such variability is a formidable challenge, and a definitive solution is still pending. While the burgeoning applications of artificial intelligence (AI) in addressing biological questions show promise, the need for *in vivo* or *in vitro* experimental validation to confirm their accuracy and biological relevance presents an ongoing and potentially never-ending challenge.

## Author contributions

RM: Conceptualization, Supervision, Writing−original draft, Writing−review and editing. GB: Conceptualization, Supervision, Writing−original draft, Writing−review & editing. SP: Writing−original draft. SD: Writing−original draft. DT: Supervision, Writing−original draft. NBM: Funding acquisition, Supervision, Writing−review and editing. EG: Conceptualization, Funding acquisition, Supervision, Writing−original draft, Writing−review and editing.

## References

[B1] Abbaszadeh-Goudarzi K. Radbakhsh S. Pourhanifeh M. H. Khanbabaei H. Davoodvandi A. Fathizadeh H. (2020). Circular RNA and Diabetes: epigenetic regulator with diagnostic role. *Curr. Mol. Med.* 20 516–526. 10.2174/1566524020666200129142106 31995005

[B2] Abuelezz N. Nasr F. AbdulKader M. Bassiouny A. Zaky A. (2021). MicroRNAs as potential orchestrators of Alzheimer’s disease-related pathologies: Insights on current status and future possibilities. *Front Aging Neurosci.* 13:743573. 10.3389/fnagi.2021.743573 34712129 PMC8546247

[B3] Adoro S. Cubillos-Ruiz J. Chen X. Deruaz M. Vrbanac V. Song M. (2015). IL-21 induces antiviral microRNA-29 in CD4 T cells to limit HIV-1 infection. *Nat Commun.* 6:7562. 10.1038/ncomms8562 PMC4481879 26108174

[B4] Akbari M. Gholipour M. Hussen B. M. Taheri M. Eslami S. Sayad A. (2022). Expression of BDNF-Associated lncRNAs in Parkinson’s disease. *Metab. Brain Dis.* 37 901–909. 10.1007/s11011-022-00946-1 35305235

[B5] Amin N. D. Bai G. Klug J. R. Bonanomi D. Pankratz M. T. Gifford W. D. (2015). Loss of motoneuron-specific microRNA-218 causes systemic neuromuscular failure. *Science* 350 1525–1529. 26680198 10.1126/science.aad2509 PMC4913787

[B6] Angot E. Brundin P. (2009). Dissecting the potential molecular mechanisms underlying alpha-synuclein cell-to-cell transfer in Parkinson’s disease. *Parkinsonism Relat. Disord.* 15 S143–S147. 10.1016/S1353-8020(09)70802-8 20082977

[B7] Arshad A. Sulaiman S. Saperi A. Jamal R. Mohamed Ibrahim N. Abdul Murad N. (2017). MicroRNAs and target genes as biomarkers for the diagnosis of early onset of Parkinson disease. *Front. Mol. Neurosci.* 10:352. 10.3389/fnmol.2017.00352 29163029 PMC5671573

[B8] Bahn J. H. Zhang Q. Li F. Chan T. M. Lin X. Kim Y. (2015). The landscape of microRNA, Piwi-interacting RNA, and circular RNA in human saliva. *Clin. Chem.* 61 221–230. 10.1373/clinchem.2014.230433 25376581 PMC4332885

[B9] Bai X. Wang J. Zhang X. Tang Y. He Y. Zhao J. (2022). Deficiency of miR-29a/b1 leads to premature aging and dopaminergic neuroprotection in mice. *Front. Mol. Neurosci.* 15:978191. 10.3389/fnmol.2022.978191 PMC9582353 36277485

[B10] Bai X. Zhang X. Fang R. Wang J. Ma Y. Liu Z. (2021). Deficiency of *miR-29b2/c* leads to accelerated aging and neuroprotection in MPTP-induced Parkinson’s disease mice. *Aging* 13 22390–22411. 34543233 10.18632/aging.203545 PMC8507277

[B11] Bai Y. Su X. Piao L. Jin Z. Jin R. (2021). Involvement of Astrocytes and microRNA Dysregulation in Neurodegenerative Diseases: From Pathogenesis to Therapeutic Potential. *Front. Mol. Neurosci.* 14:556215. 10.3389/fnmol.2021.556215 33815055 PMC8010124

[B12] Bai Y. Zhang Y. Han B. Yang L. Chen X. Huang R. (2018). Circular RNA DLGAP4 Ameliorates Ischemic Stroke Outcomes by Targeting miR-143 to Regulate Endothelial-Mesenchymal Transition Associated with Blood-Brain Barrier Integrity. *J. Neurosci.* 38 32–50. 10.1523/JNEUROSCI.1348-17.2017 29114076 PMC6705810

[B13] Barry G. (2014). Integrating the roles of long and small non-coding RNA in brain function and disease. *Mol. Psychiatry* 19 410–416. 10.1038/mp.2013.196 24468823

[B14] Bartel D. P. (2004). MicroRNAs: Genomics, biogenesis, mechanism, and function. *Cell* 116 281–297. 14744438 10.1016/s0092-8674(04)00045-5

[B15] Bohnsack J. P. Teppen T. Kyzar E. J. Dzitoyeva S. Pandey S. C. (2019). The lncRNA BDNF-AS is an epigenetic regulator in the human amygdala in early onset alcohol use disorders. *Transl. Psychiatry* 9:34. 10.1038/s41398-019-0367-z 30728347 PMC6365546

[B16] Bond A. M. Vangompel M. J. Sametsky E. A. Clark M. F. Savage J. C. Disterhoft J. F. (2009). Balanced gene regulation by an embryonic brain ncRNA is critical for adult hippocampal GABA circuitry. *Nat. Neurosci*. 12 1020–1027. 10.1038/nn.2371 19620975 PMC3203213

[B17] Braak H. Rüb U. Gai W. Del Tredici K. (2003). Idiopathic Parkinson’s disease: possible routes by which vulnerable neuronal types may be subject to neuroinvasion by an unknown pathogen. *J. Neural Transm.* 110 517–536. 10.1007/s00702-002-0808-2 12721813

[B18] Cai L. Tu L. Li T. Yang X. Ren Y. Gu R. (2020). Up-regulation of microRNA-375 ameliorates the damage of dopaminergic neurons, reduces oxidative stress and inflammation in Parkinson’s disease by inhibiting SP1. *Aging* 12 672–689. 10.18632/aging.102649 31927536 PMC6977707

[B19] Cao H. Han X. Jia Y. Zhang B. (2021). Inhibition of long non-coding RNA HOXA11-AS against neuroinflammation in Parkinson’s disease model via targeting miR-124-3p mediated FSTL1/NF-κB axis. *Aging* 13 11455–11469. 10.18632/aging.202837 33839699 PMC8109130

[B20] Caravia X. M. Fanjul V. Oliver E. Roiz-Valle D. Moran-Alvarez A. Desdin-Mico G. (2018). The microRNA-29/PGC1alpha regulatory axis is critical for metabolic control of cardiac function. *PLoS Biol.* 16 e2006247. 10.1371/journal.pbio.2006247 PMC6211751 30346946

[B21] Chang S. Su Y. Chang M. Chen J. (2021). MicroRNAs mediate precise control of spinal interneuron populations to exert delicate sensory-to-motor outputs. *Elife* 10 e63768. 10.7554/eLife.63768 PMC8075582 33787491

[B22] Chen B. J. Mills J. D. Takenaka K. Bliim N. Halliday G. M. Janitz M. (2016). Characterization of circular RNAs landscape in multiple system atrophy brain. *J. Neurochem.* 139 485–496. 10.1111/jnc.13752 27470294

[B23] Chen M. Y. Fan K. Zhao L. J. Wei J. M. Gao J. X. Li Z. F. (2021). Long non-coding RNA nuclear enriched abundant transcript 1 (NEAT1) sponges microRNA-124-3p to up-regulate phosphodiesterase 4B (PDE4B) to accelerate the progression of Parkinson’s disease. *Bioengineered* 12 708–719. 10.1080/21655979.2021.1883279 33522352 PMC8806245

[B24] Chen N. Tang J. Su Q. Chou W. C. Zheng F. Guo Z. (2021). Paraquat-induced oxidative stress regulates N6-methyladenosine (m6A) modification of circular RNAs. *Environ. Pollut.* 290:117816. 10.1016/j.envpol.2021.117816 34425375

[B25] Chen W. Schuman E. (2016). Circular RNAs in brain and other tissues: A functional enigma. *Trends Neurosci.* 39 597–604. 10.1016/j.tins.2016.06.006 27445124

[B26] Chen Y. Li Z. Chen X. Zhang S. (2021). Long non-coding RNAs: From disease code to drug role. *Acta Pharm. Sin. B.* 11 340–354. 10.1016/j.apsb.2020.10.001 33643816 PMC7893121

[B27] Cheng P. Li C. Chang Y. Tsai S. Lai Y. Chan A. (2013). miR-196a ameliorates phenotypes of Huntington disease in cell, transgenic mouse, and induced pluripotent stem cell models. *Am. J. Hum. Genet.* 93 306–312. 10.1016/j.ajhg.2013.05.025 23810380 PMC3738820

[B28] Chevillet J. Lee I. Briggs H. He Y. Wang K. (2014). Issues and prospects of microRNA-based biomarkers in blood and other body fluids. *Molecules* 19 6080–6105. 10.3390/molecules19056080 24830712 PMC6271291

[B29] Chia R. Sabir M. S. Bandres-Ciga S. Saez-Atienzar S. Reynolds R. H. Gustavsson E. (2021). Genome sequencing analysis identifies new loci associated with Lewy body dementia and provides insights into its genetic architecture. *Nat. Genet.* 53 294–303. 10.1038/s41588-021-00785-3 33589841 PMC7946812

[B30] Chmielarz P. Konovalova J. Najam S. Alter H. Piepponen T. Erfle H. (2017). Dicer and microRNAs protect adult dopamine neurons. *Cell Death Dis.* 8 e2813. 10.1038/cddis.2017.214 28542144 PMC5520729

[B31] Chodroff R. A. Goodstadt L. Sirey T. M. Oliver P. L. Davies K. E. Green E. D. (2010). Long noncoding RNA genes: Conservation of sequence and brain expression among diverse amniotes. *Genome Biol*. 11 R72. 10.1186/gb-2010-11-7-r72 20624288 PMC2926783

[B32] Cressatti M. Song W. Turk A. Garabed L. Benchaya J. Galindez C. (2019). Glial HMOX1 expression promotes central and peripheral α-synuclein dysregulation and pathogenicity in parkinsonian mice. *Glia* 67 1730–1744. 10.1002/glia.23645 31180611

[B33] D’Ambra E. Capauto D. Morlando M. (2019). Exploring the reg-ulatory role of circular RNAs in neurodegenerative disorders. *Int. J. Mol. Sci.* 20:5477. 10.3390/ijms20215477 31689888 PMC6862314

[B34] Dauer W. Przedborski S. (2003). Parkinson’s disease: mechanisms and models. *Neuron* 39 889–909. 10.1016/s0896-6273(03)00568-3 12971891

[B35] De Gregorio R. Pulcrano S. De Sanctis C. Volpicelli F. Guatteo E. von Oerthel L. (2018). miR-34b/c Regulates Wnt1 and enhances mesencephalic dopaminergic neuron differentiation. *Stem Cell Reports* 10 1237–1250. 29526736 10.1016/j.stemcr.2018.02.006 PMC5998209

[B36] Deas E. Cremades N. Angelova P. Ludtmann M. Yao Z. Chen S. (2016). Alpha-synuclein oligomers interact with metal ions to induce oxidative stress and neuronal death in Parkinson’s Disease. *Antioxid. Redox Signal* 24 376–391. 10.1089/ars.2015.6343 26564470 PMC4999647

[B37] Devi L. Raghavendran V. Prabhu B. Avadhani N. Anandatheerthavarada H. (2008). Mitochondrial import and accumulation of alpha-synuclein impair complex I in human dopaminergic neuronal cultures and Parkinson disease brain. *J. Biol. Chem.* 283 9089–9100. 10.1074/jbc.M710012200 18245082 PMC2431021

[B38] Ding X. Zhao L. Qiao H. Wu S. Wang X. (2019). Long non-coding RNA-p21 regulates MPP^+^-induced neuronal injury by targeting miR-625 and derepressing TRPM2 in SH-SY5Y cells. *Chem. Biol. Interact* 307 73–81. 10.1016/j.cbi.2019.04.017 31004593

[B39] Dong Y. Han L. Xu Z. (2018). Suppressed microRNA-96 inhibits iNOS expression and dopaminergic neuron apoptosis through inactivating the MAPK signaling pathway by targeting CACNG5 in mice with Parkinson’s disease. *Mol. Med.* 24:61. 10.1186/s10020-018-0059-9 30486773 PMC6263543

[B40] Dorostgou Z. Yadegar N. Dorostgou Z. Khorvash F. Vakili O. (2022). Novel insights into the role of circular RNAs in Parkinson disease: An emerging renaissance in the management of neurodegenerative diseases. *J. Neurosci. Res.* 100 1775–1790. 10.1002/jnr.25094 35642104

[B41] Dos Santos M. Scheller D. Schulte C. Mesa I. Colman P. Bujac S. (2018). Evaluation of cerebrospinal fluid proteins as potential biomarkers for early stage Parkinson’s disease diagnosis. *PLoS One* 13:e0206536. 10.1371/journal.pone.0206536 30383831 PMC6211693

[B42] Doxakis E. (2010). Post-transcriptional regulation of alpha-synuclein expression by mir-7 and mir-153. *J. Biol. Chem.* 285 12726–12734. 10.1074/jbc.M109.086827 20106983 PMC2857101

[B43] Doxakis E. (2022). Insights into the multifaceted role of circular RNAs: implications for Parkinson’s disease pathogenesis and diagnosis. *N. P. J. Parkinsons Dis.* 8:7. 10.1038/s41531-021-00265-9 35013342 PMC8748951

[B44] Duan Y. Wang Y. Liu Y. Jin Z. Liu C. Yu X. (2023). Circular RNAs in Parkinson’s Disease: Reliable Biological Markers and Targets for Rehabilitation. *Mol. Neurobiol.* 60 3261–3276. 10.1007/s12035-023-03268-0 36840847

[B45] Dube U. Del-Aguila J. L. Li Z. Budde J. P. Jiang S. Hsu S. (2019). An atlas of cortical circular RNA expression in Alzheimer disease brains demonstrates clinical and pathological associations. *Nat. Neurosci.* 22 1903–1912. 10.1038/s41593-019-0501-5 31591557 PMC6858549

[B46] Dunn A. Stout K. Ozawa M. Lohr K. Hoffman C. Bernstein A. (2017). Synaptic vesicle glycoprotein 2C (SV2C) modulates dopamine release and is disrupted in Parkinson disease. *Proc. Natl. Acad. Sci. U. S. A.* 114 E2253–E2262. 10.1073/pnas.1616892114 28246328 PMC5358362

[B47] Errichelli L. Dini Modigliani S. Laneve P. Colantoni A. Legnini I. Capauto D. (2017). FUS affects circular RNA expression in murine embryonic stem cell-derived motor neurons. *Nat. Commun.* 8:14741. 10.1038/ncomms14741 28358055 PMC5379105

[B48] Fagerberg L. Hallström B. M. Oksvold P. Kampf C. Djureinovic D. Odeberg J. (2014). Analysis of the human tissue-specific expression by genome-wide integration of transcriptomics and antibody-based proteomics. *Mol. Cell Proteomics* 13 397–406. 10.1074/mcp.M113.035600 24309898 PMC3916642

[B49] Fazeli S. Motovali-Bashi M. Peymani M. Hashemi M. Etemadifar M. Nasr-Esfahani M. (2020). A compound downregulation of SRRM2 and miR-27a-3p with upregulation of miR-27b-3p in PBMCs of Parkinson’s patients is associated with the early stage onset of disease. *PLoS One* 15:e0240855. 10.1371/journal.pone.0240855 33171483 PMC7654768

[B50] Feng Z. Zhang L. Wang S. Hong Q. (2020). Circular RNA circDLGAP4 exerts neuroprotective effects via modulating miR-134-5p/CREB pathway in Parkinson’s disease. *Biochem. Biophys. Res. Commun.* 522 388–394. 10.1016/j.bbrc.2019.11.102 31761328

[B51] Galvin J. Lee V. Trojanowski J. (2001). Synucleinopathies: clinical and pathological implications. *Arch. Neurol.* 58 186–190. 10.1001/archneur.58.2.186 11176955

[B52] Gascon E. Lynch K. Ruan H. Almeida S. Verheyden J. Seeley W. (2014). Alterations in microRNA-124 and AMPA receptors contribute to social behavioral deficits in frontotemporal dementia. *Nat. Med.* 20 1444–1451. 10.1038/nm.3717 25401692 PMC4257887

[B53] Gokul S. Rajanikant G. (2018). Circular RNAs in brain physiology and disease. *Adv. Exp. Med. Biol.* 1087 231–237. 10.1007/978-981-13-1426-1_18 30259370

[B54] Grasso M. Piscopo P. Talarico G. Ricci L. Crestini A. Tosto G. (2019). Plasma microRNA profiling distinguishes patients with frontotemporal dementia from healthy subjects. *Neurobiol. Aging.* 84 e1–e240. 10.1016/j.neurobiolaging.2019.01.024 30826067

[B55] Guatteo E. Berretta N. Monda V. Ledonne A. Mercuri N. (2022). Pathophysiological features of nigral dopaminergic neurons in animal models of Parkinson’s Disease. *Int. J. Mol. Sci.* 23:4508. 10.3390/ijms23094508 35562898 PMC9102081

[B56] Guatteo E. Chung K. Bowala T. Bernardi G. Mercuri N. Lipski J. (2005). Temperature sensitivity of dopaminergic neurons of the substantia nigra pars compacta: involvement of transient receptor potential channels. *J. Neurophysiol.* 94 3069–3080. 10.1152/jn.00066.2005 16014800

[B57] Guatteo E. Rizzo F. Federici M. Cordella A. Ledonne A. Latini L. (2017). Functional alterations of the dopaminergic and glutamatergic systems in spontaneous α-synuclein overexpressing rats. *Exp. Neurol.* 287 21–33. 10.1016/j.expneurol.2016.10.009 27771352

[B58] Guhathakurta S. Bok E. Evangelista B. A. Kim Y. S. (2017). Deregulation of α-synuclein in Parkinson’s disease: Insight from epigenetic structure and transcriptional regulation of SNCA. *Prog. Neurobiol*. 154 21–36. 10.1016/j.pneurobio.2017.04.004 28445713 PMC5557282

[B59] Guhathakurta S. Kim J. Adams L. Basu S. Song M. Adler E. (2021). Targeted attenuation of elevated histone marks at SNCA alleviates α-synuclein in Parkinson’s disease. *EMBO Mol. Med.* 13 e12188. 10.15252/emmm.202012188 33428332 PMC7863397

[B60] Guhathakurta S. Song M. K. Basu S. Je G. Cristovao A. C. Kim Y. S. (2022). Regulation of A lpha-Synuclein Gene (SNCA) by Epigenetic Modifier TET1 in Parkinson Disease. *Int. Neurourol. J.* 26 S85–S93. 36503211 10.5213/inj.2222206.103 PMC9767688

[B61] Han T. S. Hur K. Cho H. S. Ban H. S. (2020). Epigenetic Associations between lncRNA/circRNA and miRNA in Hepatocellular Carcinoma. *Cancers* 12:2622. 10.3390/cancers12092622 32937886 PMC7565033

[B62] Hanan M. Simchovitz A. Yayon N. Vaknine S. Cohen-Fultheim R. Karmon M. (2020). A Parkinson’s disease CircRNAs Resource reveals a link between circSLC8A1 and oxidative stress. *EMBO Mol. Med.* 12 e11942. 10.15252/emmm.201911942 32715657 PMC7507321

[B63] Hansen T. B. Wiklund E. D. Bramsen J. B. Villadsen S. B. Statham A. L. Clark S. J. (2011). miRNA-dependent gene silenc-ing involving Ago2-mediated cleavage of a circular antisense RNA. *EMBO J.* 30 4414–4422. 10.1038/emboj.2011.359 21964070 PMC3230379

[B64] Hao Z. Dang W. Zhu Q. Xu J. (2023). Long non-coding RNA UCA1 regulates MPP+-induced neuronal damage through the miR-671-5p/KPNA4 pathway in SK-N-SH cells. *Metab. Brain Dis.* 38 961–972. 10.1007/s11011-022-01118-x 36515797

[B65] Hartl M. Grunwald Kadow I. (2013). New roles for “old” microRNAs in nervous system function and disease. *Front. Mol. Neurosci.* 6:51. 10.3389/fnmol.2013.00051 24399929 PMC3871958

[B66] He L. Hannon G. J. (2004). MicroRNAs: Small RNAs with a big role in gene regulation. *Nat. Rev. Genet.* 5 522–531. 15211354 10.1038/nrg1379

[B67] Heyer M. Pani A. Smeyne R. Kenny P. Feng G. (2012). Normal midbrain dopaminergic neuron development and function in miR-133b mutant mice. *J. Neurosci.* 32 10887–10894. 22875923 10.1523/JNEUROSCI.1732-12.2012 PMC3752074

[B68] Honarmand Tamizkar K. Gorji P. Gholipour M. Hussen B. M. Mazdeh M. Eslami S. (2021). Parkinson’s Disease Is Associated With Dysregulation of Circulatory Levels of lncRNAs. *Front. Immunol.* 12:763323. 10.3389/fimmu.2021.763323 34868009 PMC8632636

[B69] Horst C. Titze-de-Almeida R. Titze-de-Almeida S. (2017). The involvement of Eag1 potassium channels and miR-34a in rotenone-induced death of dopaminergic SH-SY5Y cells. *Mol. Med. Rep.* 15 1479–1488. 10.3892/mmr.2017.6191 28259991 PMC5364983

[B70] Huang J. L. Qin M. C. Zhou Y. Xu Z. H. Yang S. M. Zhang F. (2018). Comprehensive analysis of differentially expressed profiles of Alzheimer’s disease associated circular RNAs in an Alzheimer’s disease mouse model. *Aging* 10 253–265. 10.18632/aging.101387 29448241 PMC5842852

[B71] Huang T. Liu Y. Huang M. Zhao X. Cheng L. (2010). Wnt1-cre-mediated conditional loss of Dicer results in malformation of the midbrain and cerebellum and failure of neural crest and dopaminergic differentiation in mice. *J. Mol. Cell Biol.* 2 152–163. 20457670 10.1093/jmcb/mjq008

[B72] Huang T. Zhao J. Y. Pan R. R. Jiang T. Fu X. X. Huang Q. (2023). Dysregulation of Circulatory Levels of lncRNAs in Parkinson’s Disease. *Mol. Neurobiol.* 60 317–328. 10.1007/s12035-022-03086-w 36264433

[B73] Hyun J. Choi S. Diehl A. Jung Y. (2014). Potential role of Hedgehog signaling and microRNA-29 in liver fibrosis of IKKβ-deficient mouse. *J. Mol. Histol.* 45 103–112. 23949847 10.1007/s10735-013-9532-5

[B74] Jia E. Zhou Y. Liu Z. Wang L. Ouyang T. Pan M. (2020). Transcriptomic Profiling of Circular RNA in Different Brain Regions of Parkinson’s Disease in a Mouse Model. *Int. J. Mol. Sci.* 21:3006. 10.3390/ijms21083006 32344560 PMC7216060

[B75] Junn E. Lee K. Jeong B. Chan T. Im J. Mouradian M. (2009). Repression of alpha-synuclein expression and toxicity by microRNA-7. *Proc. Natl. Acad. Sci. U. S. A.* 106 13052–13057. 10.1073/pnas.0906277106 19628698 PMC2722353

[B76] Kim J. Inoue K. Ishii J. Vanti W. Voronov S. Murchison E. (2007). MicroRNA feedback circuit in midbrain dopamine neurons. *Science* 317 1220–1224. 17761882 10.1126/science.1140481 PMC2782470

[B77] Kong F. Lv Z. Wang L. Zhang K. Cai Y. Ding Q. (2021). RNA-sequencing of peripheral blood circular RNAs in Parkinson disease. *Medicine* 100 e25888. 10.1097/MD.0000000000025888 34114985 PMC8202568

[B78] Kopp F. Mendell J. T. (2018). Functional classification and experimental dissection of long noncoding RNAs. *Cell* 172 393–407. 10.1016/j.cell.2018.01.011 29373828 PMC5978744

[B79] Kountouras J. Zavos C. Polyzos S. Deretzi G. Vardaka E. Giartza- Taxidou E. (2012). *Helicobacter* pylori infection and Parkinson’s disease: Apoptosis as an underlying com-mon contributor. *Eur. J. Neurol.* 19 e56. 10.1111/j.1468-1331.2012.03695.x 22577753

[B80] Kramer M. Schulz-Schaeffer W. (2007). Presynaptic alpha-synuclein aggregates, not Lewy bodies, cause neurodegeneration in dementia with Lewy bodies. *J. Neurosci.* 27 1405–1410. 10.1523/JNEUROSCI.4564-06.2007 17287515 PMC6673583

[B81] Kumar L. Shamsuzzama, Jadiya P. Haque R. Shukla S. Nazir A. (2018). Functional Characterization of Novel Circular RNA Molecule, circzip-2 and Its Synthesizing Gene zip-2 in C. elegans Model of Parkinson’s Disease. *Mol. Neurobiol.* 55 6914–6926. 10.1007/s12035-018-0903-5 29363043

[B82] Kwon J. J. Factora T. D. Dey S. Kota J. (2019). A systematic review of miR-29 in cancer. *Mol. Ther. Oncolytics* 12 173–194. 30788428 10.1016/j.omto.2018.12.011 PMC6369137

[B83] Kyzar E. J. Bohnsack J. P. Pandey S. C. (2022). Current and future perspectives of noncoding RNAs in brain function and neuropsychiatric disease. *Biol. Psychiatry* 91 183–193. 10.1016/j.biopsych.2021.08.013 34742545 PMC8959010

[B84] Lang Y. Zhang H. Yu H. Li Y. Liu X. Li M. (2022). Long non-coding RNA myocardial infarction-associated transcript promotes 1-Methyl-4-phenylpyridinium ion-induced neuronal inflammation and oxidative stress in Parkinson’s disease through regulating microRNA-221-3p/transforming growth factor/nuclear factor E2-related factor 2 axis. *Bioengineered* 13 930–940. 10.1080/21655979.2021.2015527 34967706 PMC8805986

[B85] Ledonne A. Massaro Cenere M. Paldino E. D’Angelo V. D’Addario S. Casadei N. (2023). Morpho-functional changes of nigral dopamine neurons in an α-synuclein model of Parkinson’s disease. *Mov Disord.* 38 256–266. 10.1002/mds.29269 36350188

[B86] Li J. Sun D. Pu W. Wang J. Peng Y. (2020). Circular RNAs in cancer: Biogenesis, function, and clinical significance. *Trends Cancer* 6 319–326. 10.1016/j.trecan.2020.01.012 32209446

[B87] Li Y. Lv Z. Zhang J. Ma Q. Li Q. Song L. (2020). Profiling of differentially expressed circular RNAs in peripheral blood mononuclear cells from Alzheimer’s disease patients. *Metab. Brain Dis.* 35 201–213. 10.1007/s11011-019-00497-y 31834549

[B88] Li Z. Lin Y. Mao L. Zhang L. (2023). Expression characteristics of circular RNA in human traumatic brain injury. *Front. Neurol.* 13:1086553. 10.3389/fneur.2022.1086553 36712438 PMC9874311

[B89] Lin H. Li Z. Chen C. Luo X. Xiao J. Dong D. (2011). Transcriptional and post-transcriptional mechanisms for oncogenic overexpression of ether à go-go K+ channel. *PLoS One* 6:e20362. 10.1371/journal.pone.0020362 21655246 PMC3105031

[B90] Lin Q. Hou S. Dai Y. Jiang N. Lin Y. (2019). LncRNA HOTAIR targets miR-126-5p to promote the progression of Parkinson’s disease through RAB3IP. *Biol. Chem.* 400 1217–1228. 10.1515/hsz-2018-0431 30738012

[B91] Liu J. Liu D. Zhao B. Jia C. Lv Y. Liao J. (2020). Long non-coding RNA NEAT1 mediates MPTP/MPP+-induced apoptosis via regulating the miR-124/KLF4 axis in Parkinson’s disease. *Open Life Sci.* 15 665–676. 10.1515/biol-2020-0069 33817255 PMC7747504

[B92] Liu Q. Li Q. Zhang R. Wang H. Li Y. Liu Z. (2022). circ-Pank1 promotes dopaminergic neuron neurodegeneration through modulating miR-7a-5p/α-syn pathway in Parkinson’s disease. *Cell Death Dis.* 13:477. 10.1038/s41419-022-04934-2 PMC9120029 35589691

[B93] Liu T. Im W. Mook-Jung I. Kim M. (2015). MicroRNA-124 slows down the progression of Huntington’s disease by promoting neurogenesis in the striatum. *Neural Regen. Res.* 10 786–791. 10.4103/1673-5374.156978 26109954 PMC4468771

[B94] Liu W. Li L. Liu S. Wang Z. Kuang H. Xia Y. (2019). MicroRNA Expression Profiling Screen miR-3557/324-Targeted *CaMK/mTOR* in the Rat Striatum of Parkinson’s Disease in Regular Aerobic Exercise. *Biomed. Res. Int.* 2019:7654798. 10.1155/2019/7654798 31309116 PMC6594308

[B95] Liu W. Zhang Q. Zhang J. Pan W. Zhao J. Xu Y. (2017). Long non-coding RNA MALAT1 contributes to cell apoptosis by sponging miR-124 in Parkinson disease. *Cell Biosci.* 7:19. 10.1186/s13578-017-0147-5 28439401 PMC5401610

[B96] Lu M. (2020). Circular RNA: Functions, applications and prospects. *ExRNA* 2 1–7. 10.1186/s41544-019-0046-5

[B97] Lu S. Yang X. Wang C. Chen S. Lu S. Yan W. (2019). Current status and potential role of circular RNAs in neurological disorders. *J. Neurochem.* 150 237–248. 10.1111/jnc.14724 31099046

[B98] Luk K. Kehm V. Zhang B. O’Brien P. Trojanowski J. Lee V. (2012). Intracerebral inoculation of pathological α-synuclein initiates a rapidly progressive neurodegenerative α-synucleinopathy in mice. *J. Exp. Med.* 209 975–986. 10.1084/jem.20112457 22508839 PMC3348112

[B99] Lukiw W. J. (2013). Circular RNA (circRNA) in Alzheimer’s disease (AD). *Front. Genet.* 4:307. 10.3389/fgene.2013.00307 24427167 PMC3875874

[B100] Lv K. Liu Y. Zheng Y. Dai S. Yin P. Miao H. (2021). Long non-coding RNA MALAT1 regulates cell proliferation and apoptosis via miR-135b-5p/GPNMB axis in Parkinson’s disease cell model. *Biol. Res.* 54:10. 10.1186/s40659-021-00332-8 33726823 PMC7968316

[B101] Ma J. Sun W. Chen S. Wang Z. Zheng J. Shi X. (2022). The long noncoding RNA GAS5 potentiates neuronal injury in Parkinson’s disease by binding to microRNA-150 to regulate Fosl1 expression. *Exp. Neurol.* 347:113904. 10.1016/j.expneurol.2021.113904 34755654

[B102] Ma X. Zhang H. Yin H. Geng S. Liu Y. Liu C. (2021). Up-regulated microRNA-218-5p ameliorates the damage of dopaminergic neurons in rats with Parkinson’s disease via suppression of LASP1. *Brain Res. Bull.* 166 92–101. 10.1016/j.brainresbull.2020.10.019 33144090

[B103] Mahmoudi E. Cairns M. J. (2019). Circular RNAs are temporospatially regulated throughout development and ageing in the rat. *Sci. Rep.* 9:2564. 10.1038/s41598-019-38860-9 30796328 PMC6385508

[B104] Mahmoudi E. Green M. J. Cairns M. J. (2021). Dysregulation of circRNA expression in the peripheral blood of individuals with schizophrenia and bipolar disorder. *J. Mol. Med.* 99 981–991. 10.1007/s00109-021-02070-6 33782720

[B105] Mahul-Mellier A. Burtscher J. Maharjan N. Weerens L. Croisier M. Kuttler F. (2020). The process of Lewy body formation, rather than simply α-synuclein fibrillization, is one of the major drivers of neurodegeneration. *Proc. Natl. Acad. Sci. U. S. A.* 117 4971–4982. 10.1073/pnas.1913904117 32075919 PMC7060668

[B106] Martin K. Ephrussi A. (2009). mRNA localization: gene expression in the spatial dimension. *Cell* 136 719–730. 19239891 10.1016/j.cell.2009.01.044 PMC2819924

[B107] Mayo M. Bordelon Y. (2014). Dementia with Lewy bodies. *Semin. Neurol.* 34 182–188. 24963677 10.1055/s-0034-1381741

[B108] Mazzeo F. Meccariello R. Guatteo E. (2023). Molecular and Epigenetic Aspects of Opioid Receptors in Drug Addiction and Pain Management in Sport. *Int. J. Mol. Sci.* 24:7831. 10.3390/ijms24097831 37175536 PMC10178540

[B109] McMillan K. J. Murray T. K. Bengoa-Vergniory N. Cordero-Llana O. Cooper J. Buckley A. (2017). Loss of MicroRNA-7 Regulation Leads to α-Synuclein Accumulation and Dopaminergic Neuronal Loss In Vivo. *Mol. Ther.* 25 2404–2414. 10.1016/j.ymthe.2017.08.017 28927576 PMC5628933

[B110] Mehta S. L. Dempsey R. J. Vemuganti R. (2020). Role of circular RNAs in brain development and CNS diseases. *Prog. Neurobiol.* 186:101746. 10.1016/j.pneurobio.2020.101746 31931031 PMC7024016

[B111] Modarresi F. Faghihi M. A. Lopez-Toledano M. A. Fatemi R. P. Magistri M. Brothers S. P. (2012). Inhibition of natural antisense transcripts in vivo results in gene-specific transcriptional upregulation. *Nat. Biotechnol*. 30 453–459. 10.1038/nbt.2158 22446693 PMC4144683

[B112] Nakamori M. Junn E. Mochizuki H. Mouradian M. (2019). Nucleic Acid-Based Therapeutics for Parkinson’s Disease. *Neurotherapeutics* 16 287–298. 10.1007/s13311-019-00714-7 30756362 PMC6554378

[B113] Nakamura K. Nemani V. Wallender E. Kaehlcke K. Ott M. Edwards R. (2008). Optical reporters for the conformation of alpha-synuclein reveal a specific interaction with mitochondria. *J. Neurosci.* 28 12305–12317. 10.1523/JNEUROSCI.3088-08.2008 19020024 PMC6671709

[B114] Neal M. Richardson J. (2018). Epigenetic regulation of astrocyte function in neuroinflammation and neurodegeneration. *Biochim. Biophys. Acta Mol. Basis Dis.* 1864 432–443. 10.1016/j.bbadis.2017.11.004 29113750 PMC5743548

[B115] Pang X. Hogan E. M. Casserly A. Gao G. Gardner P. D. Tapper A. R. (2014). Dicer expression is essential for adult midbrain dopaminergic neuron maintenance and survival. *Mol. Cell. Neurosci.* 58 22–28. 24184162 10.1016/j.mcn.2013.10.009 PMC3944994

[B116] Peng L. Yuan X. Q. Li G. C. (2015). The emerging landscape of circu-lar RNA ciRS-7 in cancer. *Oncol. Rep.* 33 2669–2674. 10.3892/or.2015.3904 25873049

[B117] Piwecka M. Glažar P. Hernandez-Miranda L. R. Memczak S. Wolf S. A. Rybak-Wolf A. (2017). Loss of a mammalian circular RNA locus causes miRNA deregulation and affects brain function. *Science* 357 eaam8526. 10.1126/science.aam8526 28798046

[B118] Pogribny I. (2018). MicroRNAs as biomarkers for clinical studies. *Exp. Biol. Med.* 243 283–290. 10.1177/1535370217731291 28914096 PMC5813862

[B119] Ponjavic J. Oliver P. L. Lunter G. Ponting C. P. (2009). Genomic and transcriptional co-localization of protein-coding and long non-coding RNA pairs in the developing brain. *PLoS Genet*. 5:e1000617. 10.1371/journal.pgen.1000617 19696892 PMC2722021

[B120] Pukkila-Worley R. Feinbaum R. Kirienko N. V. Larkins-Ford J. Conery A. L. Ausubel F. M. (2012). Stimulation of host immune defenses by a small molecule protects C. elegans from bacterial infection. *PLoS Genet.* 8:e1002733. 10.1371/journal.pgen.1002733 22719261 PMC3375230

[B121] Pulcrano S. De Gregorio R. De Sanctis C. Volpicelli F. Piscitelli R. Speranza L. (2023). miR-218 promotes dopaminergic differentiation and controls neuron excitability and neurotransmitter release through the regulation of a synaptic-related genes network. *J. Neurosci.* 43 8104–8125. 37816598 10.1523/JNEUROSCI.0431-23.2023 PMC10697421

[B122] Qian H. Kang X. Hu J. Zhang D. Liang Z. Meng F. (2020). Author Correction: Reversing a model of Parkinson’s disease with in situ converted nigral neurons. *Nature* 584 E17. 10.1038/s41586-020-2583-3 32724206

[B123] Quan Y. Wang J. Wang S. Zhao J. (2020). Association of the Plasma Long Non-coding RNA MEG3 With Parkinson’s Disease. *Front. Neurol.* 11:532891. 10.3389/fneur.2020.532891 33329296 PMC7732627

[B124] Ravanidis S. Bougea A. Karampatsi D. Papagiannakis N. Maniati M. Stefanis L. (2021). Differentially expressed circular RNAs in peripheral blood mononuclear cells of patients with Parkinson’s Disease. *Mov. Disord.* 36 1170–1179. 10.1002/mds.28467 33433033 PMC8248110

[B125] Reed E. Latourelle J. Bockholt J. Bregu J. Smock J. Paulsen J. (2018). MicroRNAs in CSF as prodromal biomarkers for Huntington disease in the PREDICT-HD study. *Neurology* 90 e264–e272. 10.1212/WNL.0000000000004844 29282329 PMC5798654

[B126] Reichenstein I. Eitan C. Diaz-Garcia S. Haim G. Magen I. Siany A. (2019). Human genetics and neuropathology suggest a link between miR-218 and amyotrophic lateral sclerosis pathophysiology. *Sci. Transl. Med.* 11 eaav5264–eaav5224. 31852800 10.1126/scitranslmed.aav5264 PMC7057809

[B127] Rey F. Pandini C. Messa L. Launi R. Barzaghini B. Zangaglia R. (2021). α- Synuclein antisense transcript SNCA-AS1 regulates synapses- and aging-related genes suggesting its implication in Parkinson’s disease. *Aging Cell.* 20 e13504. 10.1111/acel.13504 34799977 PMC8672788

[B128] Rivetti di Val Cervo P. Romanov R. Spigolon G. Masini D. Martín-Montañez E. Toledo E. (2017). Induction of functional dopamine neurons from human astrocytes in vitro and mouse astrocytes in a Parkinson’s disease model. *Nat. Biotechnol.* 35 444–452. 28398344 10.1038/nbt.3835

[B129] Ruffo P. De Amicis F. Giardina E. Conforti F. L. (2023). Long-noncoding RNAs as epigenetic regulators in neurodegenerative diseases. *Neural Regen. Res.* 18 1243–1248. 10.4103/1673-5374.358615 36453400 PMC9838156

[B130] Rybak-Wolf A. Stottmeister C. Glažar P. Jensm M. Pino N. Giusti S. (2015). Circular RNAs in the mammalian brain are highly abundant, conserved, and dynamically expressed. *Mol. Cell.* 58 870–885. 10.1016/j.molcel.2015.03.027 25921068

[B131] Sang Q. Liu X. Wang L. Qi L. Sun W. Wang W. (2018). CircSNCA downregulation by pramipexole treatment mediates cell apoptosis and autophagy in Parkinson’s disease by targeting miR-7. *Aging* 10 1281–1293. 10.18632/aging.101466 29953413 PMC6046232

[B132] Sarıekiz F. G. Tomatır A. G. Tokgün P. E. Bir L. S. (2023). Evaluation of Long Non-coding RNA Expression Profiles in Peripheral Blood Mononuclear Cells of Patients with Parkinson’s Disease. *Mol. Neurobiol.* 10.1007/s12035-023-03470-0 [Epub ahead of print]. 37436601

[B133] Sasaki K. Doh-ura K. Wakisaka Y. Iwaki T. (2002). Clusterin/apolipoprotein J is associated with cortical Lewy bodies: immunohistochemical study in cases with alpha-synucleinopathies. *Acta Neuropathol.* 104 225–230. 10.1007/s00401-002-0546-4 12172907

[B134] Sati S. Ghosh S. Jain V. Scaria V. Sengupta S. (2012). Genome-wide analysis reveals distinct patterns of epigenetic features in long noncoding RNA loci. *Nucleic Acids Res*. 40 10018–10100. 10.1093/nar/gks776 22923516 PMC3488231

[B135] Schaffner S. Kobor M. (2022). DNA methylation as a mediator of genetic and environmental influences on Parkinson’s disease susceptibility: Impacts of alpha-Synuclein, physical activity, and pesticide exposure on the epigenome. *Front. Genet.* 13:971298. 10.3389/fgene.2022.971298 36061205 PMC9437223

[B136] Schirinzi T. Madeo G. Martella G. Maltese M. Picconi B. Calabresi P. (2016). Early synaptic dysfunction in Parkinson’s disease: Insights from animal models. *Mov. Disord.* 31 802–813. 10.1002/mds.26620 27193205

[B137] Schulz D. Baines R. Hempel C. Li L. Liss B. Misonou H. (2006). Cellular excitability and the regulation of functional neuronal identity: from gene expression to neuromodulation. *J. Neurosci.* 26 10362–10367. 10.1523/JNEUROSCI.3194-06.2006 17035518 PMC6674680

[B138] Shavali S. Brown-Borg H. Ebadi M. Porter J. (2008). Mitochondrial localization of alpha-synuclein protein in alpha-synuclein overexpressing cells. *Neurosci. Lett.* 439 125–128. 10.1016/j.neulet.2008.05.005 18514418 PMC2502066

[B139] Sherazi S. A. M. Abbasi A. Jamil A. Uzair M. Ikram A. Qamar S. (2023). Molecular hallmarks of long non-coding RNAs in aging and its significant effect on aging-associated diseases. *Neural Regeny. Res*. 18 959–968. 10.4103/1673-5374.355751 36254975 PMC9827784

[B140] Singh A. Mewes K. Gross R. DeLong M. Obeso J. Papa S. (2016). Human striatal recordings reveal abnormal discharge of projection neurons in Parkinson’s disease. *Proc. Natl. Acad. Sci. U. S. A.* 113 9629–9634. 10.1073/pnas.1606792113 27503874 PMC5003232

[B141] Sivagurunathan N. Ambatt A. T. S. Calivarathan L. (2022). Role of Long Non-coding RNAs in the Pathogenesis of Alzheimer’s and Parkinson’s Diseases. *Curr. Aging Sci*. 15 84–96. 10.2174/1874609815666220126095847 35081899

[B142] Srivastava A. Dada O. Qian J. Al-Chalabi N. Fatemi A. B. Gerretsen P. (2021). Epigenetics of Schizophrenia. *Psychiatry Res.* 305:114218. 10.1016/j.psychres.2021.114218 34638051

[B143] Stein C. McLendon J. Witmer N. Boudreau R. (2022). Modulation of miR-181 influences dopaminergic neuronal degeneration in a mouse model of Parkinson’s disease. *Mol. Ther. Nucleic Acids* 28 1–15. 10.1016/j.omtn.2022.02.007 35280925 PMC8899134

[B144] Steiner J. Quansah E. Brundin P. (2018). The concept of alpha-synuclein as a prion-like protein: ten years after. *Cell Tissue Res.* 373 161–173. 10.1007/s00441-018-2814-1 29480459 PMC6541204

[B145] Subramaniam S. Vergnes L. Franich N. Reue K. Chesselet M. (2014). Region specific mitochondrial impairment in mice with widespread overexpression of alpha-synuclein. *Neurobiol. Dis.* 70 204–213. 10.1016/j.nbd.2014.06.017 25016198 PMC4205109

[B146] Sun Y. Sukumaran P. Selvaraj S. Cilz N. Schaar A. Lei S. (2018). TRPM2 Promotes Neurotoxin MPP^+^/MPTP-Induced Cell Death. *Mol. Neurobiol.* 55 409–420. 10.1007/s12035-016-0338-9 27957685 PMC5468501

[B147] Szego É Dominguez-Meijide A. Gerhardt E. König A. Koss D. Li W. (2019). Cytosolic trapping of a mitochondrial heat shock protein is an early pathological event in synucleinopathies. *Cell Rep.* 28 65–77.e1. 10.1016/j.celrep.2019.06.009 31269451

[B148] Szelągowski A. Kozakiewicz M. A. (2023). Glance at Biogenesis and Functionality of MicroRNAs and Their Role in the Neuropathogenesis of Parkinson’s Disease. *Oxid. Med. Cell Longev.* 2023:7759053. 10.1155/2023/7759053 PMC10270766 37333462

[B149] Tan C. Plotkin J. Venø M. von Schimmelmann M. Feinberg P. Mann S. (2013). MicroRNA-128 governs neuronal excitability and motor behavior in mice. *Science* 342 1254–1258. 10.1126/science.1244193 24311694 PMC3932786

[B150] Tan G. Wang L. Liu Y. Zhang H. Feng W. Liu Z. (2021). The alterations of circular RNA expression in plasma exosomes from patients with schizophrenia. *J. Cell. Physiol.* 236 458–467. 10.1002/jcp.29873 32542700

[B151] Tatura R. Kraus T. Giese A. Arzberger T. Buchholz M. Höglinger G. (2016). Parkinson’s disease: SNCA-, PARK2-, and LRRK2- targeting microRNAs elevated in cingulate gyrus. *Parkinsonism Relat. Disord.* 33 115–121. 10.1016/j.parkreldis.2016.09.028 27717584

[B152] Tozzi A. Sciaccaluga M. Loffredo V. Megaro A. Ledonne A. Cardinale A. (2021). Dopamine-dependent early synaptic and motor dysfunctions induced by α-synuclein in the nigrostriatal circuit. *Brain* 144 3477–3491. 10.1093/brain/awab242 34297092 PMC8677552

[B153] Traber G. Yu A. (2023). RNAi-Based Therapeutics and Novel RNA Bioengineering Technologies. *J. Pharmacol. Exp. Ther.* 384 133–154. 10.1124/jpet.122.001234 35680378 PMC9827509

[B154] Uwatoko H. Hama Y. Iwata I. T. Shirai S. Matsushima M. Yabe I. (2019). Identification of plasma microRNA expression changes in multiple system atrophy and Parkinson’s dis-ease. *Mol. Brain* 12 1–10. 10.1186/s13041-019-0471-2 31088501 PMC6518614

[B155] Vilaça-Faria H. Salgado A. Teixeira F. (2019). Mesenchymal Stem Cells-derived Exosomes: A New Possible Therapeutic Strategy for Parkinson’s Disease? *Cells* 8:118. 10.3390/cells8020118 30717429 PMC6406999

[B156] Vogel A. Upadhya R. Shetty A. K. (2018). Neural stem cell derived extracellular vesicles: Attributes and prospects for treating neuro-degenerative disorders. *eBioMedicine* 38 273–282. 10.1016/j.ebiom.2018.11.026 30472088 PMC6306394

[B157] Wan Z. Rasheed M. Li Y. Li Q. Wang P. Li J. (2023). miR-218-5p and miR-320a-5p as biomarkers for brain disorders: focus on the major depressive disorder and Parkinson’s disease. *Mol. Neurobiol.* 10.1007/s12035-023-03391-y [Epub ahead of print]. 37329382

[B158] Wang H. Zhang M. Wei T. Zhou J. Zhang Y. Guo D. (2021). Long non-coding RNA SNHG1 mediates neuronal damage in Parkinson’s disease model cells by regulating miR-216a-3p/Bcl-2-associated X protein. *Ann. Transl. Med.* 9:851. 10.21037/atm-21-1613 34164485 PMC8184415

[B159] Wang J. Chen J. Sen S. (2016). MicroRNA as biomarkers and diagnostics. *J. Cell Physiol.* 231 25–30. 10.1002/jcp.25056 26031493 PMC8776330

[B160] Wang S. W. Liu Z. Shi Z. S. (2018). Non-Coding RNA in Acute Ischemic Stroke: Mechanisms, Biomarkers and Therapeutic Targets. *Cell Transplant.* 27 1763–1777. 10.1177/0963689718806818 30362372 PMC6300774

[B161] Wang W. Lv R. Zhang J. Liu Y. (2021). circSAMD4A participates in the apoptosis and autophagy of dopaminergic neurons via the miR-29c-3p-mediated AMPK/mTOR pathway in Parkinson’s disease. *Mol. Med. Rep.* 24:540. 10.3892/mmr.2021.12179 34080649 PMC8170871

[B162] Wang X. Zhao B. S. Roundtree I. A. Lu Z. Han D. Ma H. (2015). N(6)-methyladenosine modulates messenger RNA translation efficiency. *Cell* 161 1388–1399. 10.1016/j.cell.2015.05.014 26046440 PMC4825696

[B163] Xin C. Liu J. (2021). Long non-coding RNAs in Parkinson’s disease. *Neurochem. Res.* 46 1031–1042. 10.1007/s11064-021-03230-3 33544326

[B164] Xing R. Li L. Liu X. Tian B. Cheng Y. (2020). Down regulation of miR-218, miR-124, and miR-144 relates to Parkinson’s disease via activating NF-κB signaling. *Kaohsiung J. Med. Sci.* 36 786–792. 10.1002/kjm2.12241 32492291 PMC11896404

[B165] Xu H. Guo S. Li W. Yu P. (2015). The circular RNA Cdr1as, via miR-7 and its targets, regulates insulin transcription and secretion in islet cells. *Sci. Rep.* 5 1–12. 10.1038/srep12453 26211738 PMC4515639

[B166] Xu K. Chen D. Wang Z. Ma J. Zhou J. Chen N. (2018). Annotation and functional clustering of circRNA expression in rhesus macaque brain during aging. *Cell. Discov.* 4:48. 10.1038/s41421-018-0050-1 30245844 PMC6141548

[B167] Xylaki M. Paiva I. Al-Azzani M. Gerhardt E. Jain G. Islam M. (2023). miR-101a-3p impairs synaptic plasticity and contributes to synucleinopathy. *J. Parkinsons Dis.* 13 179–196. 10.3233/JPD-225055 36744345 PMC10041420

[B168] Yang C. Zhang Z. Zhang L. Rui H. (2016). Neuroprotective Role of MicroRNA-22 in a 6-hydroxydopamine-induced cell model of Parkinson’s disease via regulation of its target gene TRPM7. *J. Mol. Neurosci.* 60 445–452. 10.1007/s12031-016-0828-2 27631550

[B169] Yang K. Zeng L. Ge A. Wang S. Zeng J. Yuan X. (2022). A systematic review of the research progress of non-coding RNA in neuroinflammation and immune regulation in cerebral infarction/ischemia-reperfusion injury. *Front. Immunol*. 13:930171. 10.3389/fimmu.2022.930171 36275741 PMC9585453

[B170] Yang L. Wilusz J. E. Chen L. L. (2022). Biogenesis and Regulatory Roles of Circular RNAs. *Annu. Rev. Cell. Dev Biol.* 38 263–289. 10.1146/annurev-cellbio-120420-125117 35609906 PMC10119891

[B171] Yang P. Chen W. Lee J. Lin C. Chen Y. Lin C. (2023). Coumarin-chalcone hybrid LM-021 and indole derivative NC009-1 targeting inflammation and oxidative stress to protect BE(2)-M17 cells against α-synuclein toxicity. *Aging* 15 8061–8089. 10.18632/aging.204954 37578928 PMC10497001

[B172] Yang S. Lim K. H. Kim S. H. Joo J. Y. (2021). Molecular landscape of long noncoding RNAs in brain disorders. *Mol. Psychiatry* 26 1060–1074. 10.1038/s41380-020-00947-5 33173194

[B173] Yang Y. Fan X. Mao M. Song X. Wu P. Zhang Y. (2017). Extensive translation of circular RNAs driven by N6-methyladenosine. *Cell. Res.* 27 626–641. 10.1038/cr.2017.31 28281539 PMC5520850

[B174] Yao Y. Zhao Z. Zhang F. Miao N. Wang N. Xu X. (2023). microRNA-221 rescues the loss of dopaminergic neurons in a mouse model of Parkinson’s disease. *Brain Behav.* 13 e2921. 10.1002/brb3.2921 36795044 PMC10013949

[B175] Yuan X. Wu Y. Lu L. Feng J. (2022). Long noncoding RNA SNHG14 knockdown exerts a neuroprotective role in MPP+-induced Parkinson’s disease cell model through mediating miR-135b-5p/KPNA4 axis. *Metab. Brain Dis.* 37 2363–2373. 10.1007/s11011-022-01038-w 35781593

[B176] Zhai K. Liu B. Gao L. (2020). Long-Noncoding RNA TUG1 Promotes Parkinson’s Disease via Modulating MiR-152-3p/PTEN Pathway. *Hum. Gene Ther.* 31 1274–1287. 10.1089/hum.2020.106 32808542

[B177] Zhang H. Wang Z. Hu K. Liu H. (2021). Downregulation of long noncoding RNA SNHG7 protects against inflammation and apoptosis in Parkinson’s disease model by targeting the miR-425-5p/TRAF5/NF-κB axis. *J. Biochem. Mol. Toxicol.* 35 e22867. 10.1002/jbt.22867 34369042

[B178] Zhang H. Yao L. Zheng Z. Koc S. Lu G. (2022). The Role of Non-Coding RNAs in the Pathogenesis of Parkinson’s Disease: Recent Advancement. *Pharmaceuticals* 15:811. 10.3390/ph15070811 35890110 PMC9315906

[B179] Zhang M. Xin Y. (2018). Circular RNAs: A new frontier for cancer diagnosis and therapy. *J. Hematol. Oncol.* 11 1–9. 10.1186/s13045-018-0569-5 29433541 PMC5809913

[B180] Zhang N. Tang Z. Liu C. (2008). alpha-Synuclein protofibrils inhibit 26 S proteasome-mediated protein degradation: understanding the cytotoxicity of protein protofibrils in neurodegenerative disease pathogenesis. *J. Biol. Chem.* 283 20288–20298. 10.1074/jbc.M710560200 18502751

[B181] Zhao J. Zhou Y. Guo M. Yue D. Chen C. Liang G. (2020). MicroRNA-7: expression and function in brain physiological and pathological processes. *Cell Biosci.* 10:77. 10.1186/s13578-020-00436-w 32537124 PMC7288475

[B182] Zhao Y. Xie Y. Yao W. Y. Wang Y. Y. Song N. (2022). Long non-coding RNA Opa interacting protein 5-antisense RNA 1 promotes mitochondrial autophagy and protects SH-SY5Y cells from 1-methyl-4-phenylpyridine-induced damage by binding to microRNA-137 and upregulating NIX. *Kaohsiung J. Med. Sci.* 38 207–217. 10.1002/kjm2.12485 35049152 PMC11896267

[B183] Zhong L. Ju K. Chen A. Cao H. (2021). Circulating CircRNAs panel acts as a biomarker for the early diagnosis and severity of Parkinson’s disease. *Front. Aging Neurosci.* 13:684289. 10.3389/fnagi.2021.684289 34276342 PMC8281126

[B184] Zhou C. Molinie B. Daneshvar K. Pondick J. V. Wang J. Van Wittenberghe N. (2017). Genome-Wide Maps of m6A circRNAs Identify Widespread and Cell-Type-Specific Methylation Patterns that Are Distinct from mRNAs. *Cell. Rep.* 20 2262–2276. 10.1016/j.celrep.2017.08.027 28854373 PMC5705222

[B185] Zhou S. Zhang D. Guo J. Chen Z. Chen Y. Zhang J. (2020). Long non-coding RNA NORAD functions as a microRNA-204-5p sponge to repress the progression of Parkinson’s disease in vitro by increasing the solute carrier family 5 member 3 expression. *IUBMB Life.* 72 2045–2055. 10.1002/iub.2344 32687247

[B186] Zhou Y. Zhu J. Lv Y. Song C. Ding J. Xiao M. (2018). Kir6.2 Deficiency Promotes Mesencephalic Neural Precursor Cell Differentiation via Regulating miR-133b/GDNF in a Parkinson’s Disease Mouse Model. *Mol. Neurobiol*. 55, 8550–8562. 10.1007/s12035-018-1005-0 29564810

[B187] Zhou W. Y. Cai Z. R. Liu J. Wang D. S. Ju H. Q. Xu R. H. (2020). Circular RNA: metabolism, functions and interactions with proteins. *Mol. Cancer.* 19:172. 10.1186/s12943-020-01286-3 33317550 PMC7734744

[B188] Zou J. Guo Y. Wei L. Yu F. Yu B. Xu A. (2020). Long Noncoding RNA POU3F3 and α-Synuclein in Plasma L1CAM Exosomes Combined with β-Glucocerebrosidase Activity: Potential Predictors of Parkinson’s Disease. *Neurotherapeutics.* 17 1104–1119. 10.1007/s13311-020-00842-5 32236821 PMC7609611

[B189] Zucchelli S. Fedele S. Vatta P. Calligaris R. Heutink P. Rizzu P. (2019). Antisense transcription in loci associated to hereditary neurodegenerative diseases. *Mol. Neurobiol.* 56 5392–5415. 30610612 10.1007/s12035-018-1465-2 PMC6614138

